# Comparative Genomic Analysis Reveals the Metabolism and Evolution of the Thermophilic Archaeal Genus *Metallosphaera*

**DOI:** 10.3389/fmicb.2020.01192

**Published:** 2020-06-19

**Authors:** Pei Wang, Liang Zhi Li, Ya Ling Qin, Zong Lin Liang, Xiu Tong Li, Hua Qun Yin, Li Jun Liu, Shuang-Jiang Liu, Cheng-Ying Jiang

**Affiliations:** ^1^State Key Laboratory of Microbial Resources, Institute of Microbiology, Chinese Academy of Sciences, Beijing, China; ^2^College of Life Sciences, University of Chinese Academy of Sciences, Beijing, China; ^3^School of Minerals Processing and Bioengineering, Central South University, Changsha, China; ^4^Key Laboratory of Biometallurgy of Ministry of Education, Central South University, Changsha, China; ^5^Department of Pathogen Biology, School of Basic Medical Science, Xi’an Medical University, Xi’an, China

**Keywords:** comparative genomics, *Metallosphaera*, metabolic potential, evolution, horizontal gene transfer

## Abstract

Members of the genus *Metallosphaera* are widely found in sulfur-rich and metal-laden environments, but their physiological and ecological roles remain poorly understood. Here, we sequenced *Metallosphaera tengchongensis* Ric-A, a strain isolated from the Tengchong hot spring in Yunnan Province, China, and performed a comparative genome analysis with other *Metallosphaera* genomes. The genome of *M. tengchongensis* had an average nucleotide identity (ANI) of approximately 70% to that of *Metallosphaera cuprina*. Genes *sqr*, *tth*, *sir*, *tqo*, *hdr*, *tst*, *soe*, and *sdo* associated with sulfur oxidation, and gene clusters *fox* and *cbs* involved in iron oxidation existed in all *Metallosphaera* genomes. However, the adenosine-5′-phosphosulfate (APS) pathway was only detected in *Metallosphaera sedula* and *Metallosphaera yellowstonensis*, and several subunits of *fox* cluster were lost in *M. cuprina*. The complete 3-hydroxypropionate/4-hydroxybutyrate cycle and dicarboxylate/4-hydroxybutyrate cycle involved in carbon fixation were found in all *Metallosphaera* genomes. A large number of gene family gain events occurred in *M. yellowstonensis* and *M. sedula*, whereas gene family loss events occurred frequently in *M. cuprina.* Pervasive strong purifying selection was found acting on the gene families of *Metallosphaera*, of which transcription-related genes underwent the strongest purifying selection. In contrast, genes related to prophages, transposons, and defense mechanisms were under weaker purifying pressure. Taken together, this study expands knowledge of the genomic traits of *Metallosphaera* species and sheds light on their evolution.

## Introduction

Extremely acidophilic archaea of the genus *Metallosphaera* belong to the order Sulfolobales within the Crenarchaeota. Together with the genera *Acidianus* and *Sulfolobus*, physiologically versatile *Metallosphaera* spp. contribute significantly to biogeochemical element cycling and biomining processes. *Metallosphaera* spp. stand out due to their ability for facultative autotrophic growth and tolerance to high concentrations of metal ions (Ai et al., 2016; Wheaton et al., 2016). Members of the genus *Metallosphaera* grow aerobically at low pH values (<4) and high temperatures (>60°C). They are found in sulfur-rich and metal-laden environments including solfataric areas (*Metallosphaera sedula* DSM 5348 and strains from laboratory evolution) (Huber et al., 1989; Ai et al., 2016, 2017), hot springs (such as *Metallosphaera hakonensis* HO1-1 = JCM8857, *Metallosphaera cuprina* Ar-4, *Metallosphaera tengchongensis* Ric-A, *Metallosphaera yellowstonensis* MK-1, *Metallosphaera* sp. UBA165, and *Metallosphaera* sp. Obs4) (Takayanagi et al., 1996; Kozubal et al., 2008, 2011; Liu et al., 2011a; Peng et al., 2015; Parks et al., 2017), and bioleaching-related heaps (*Metallosphaera prunae* Ron 12/II) (Fuchs et al., 1995). *Metallosphaera* species are of great potential in the extraction of base and precious metals from ores exploiting their ability to oxidize reduced inorganic sulfur compounds (RISCs) and ferrous ion, a process mediated by a set of terminal oxidases that are attached to their cell membranes (Auernik and Kelly, 2008, 2010; Orell et al., 2010). Previous studies showed that application of thermophilic archaea in chalcopyrite bioleaching could achieve faster dissolution rates and higher copper leaching yields in comparison to widely used mesophilic bioleaching bacteria because the formation of the surface passivation and diffusion layer of chalcopyrite was significantly reduced at high temperature (Rawlings, 2005; Urbieta et al., 2015; Castro and Donati, 2016).

*Metallosphaera* species can grow on peptides in a heterotrophic mode, fix carbon dioxide using reduced sulfur compounds as reductant in an autotrophic mode, or grow on casamino acids and FeSO4 or metal sulfides in a mixotrophic mode (Peeples and Kelly, 1995; Ulrike et al., 2005; Alber et al., 2006; Auernik and Kelly, 2008, 2010; Michael et al., 2010; Han and Kelly, 2015). Autotrophic carbon fixation through the 3-hydroxypropionate/4-hydroxybutyrate (HP/HB) cycle in *Metallosphaera* has been confirmed by genomics, transcriptomics, proteomics, and biochemical assays (Sebastian et al., 2011; Jiang et al., 2014). Reduced Fe and S (Fe^2+^, HS^–^, S^0^, S_4_O_6_^2+^, and S_2_O_3_^2+^) are important electron donors for iron or sulfur-oxidizing microorganism. Unlike Fe^2+^ oxidation by the bacteria *Acidithiobacillus ferrooxidans* and *Leptospirillum ferrooxidans*, which require the blue copper protein rusticyanin and various c-type cytochromes (Rohwerder et al., 2003; Singer et al., 2008), the Fe oxidation mechanism of iron-oxidizing archaea is yet unconfirmed, although potential proteins were noticed in the genomes of *Ferroplasma*, *Sulfolobus*, and *Metallosphaera* species (Dopson et al., 2005; Bathe and Norris, 2007; Auernik et al., 2008). The genes involved in iron oxidation (Kozubal et al., 2011) such as *fox*, *cbs*, rusticyanin, and sulfocyanin have been detected in the genomes of *M. sedula* and *M. yellowstonensis*, but their pervasiveness in *Metallosphaera* is yet unknown. RISC oxidation mechanisms are complex and diverse in extremely thermoacidophilic archaea. Sulfur oxygenase reductase genes are present in the genus *Acidianus*, in the species *Sulfolobus tokadaii*, and in bacteria, but are absent from the genomes of *M. sedula* and *M. cuprina* (Auernik et al., 2008; Liu et al., 2011b), which raised the question how sulfur oxidation is initiated in *Metallosphaera*. Of the species within the genus *Metallosphaera*, genome sequences and genomic analyses have been reported for four isolates (*M. cuprina* Ar-4, *M. hakonensis* HO1-1 = JCM_8857, *M. sedula* DSM_5348, and *M. yellowstonensis* MK1) (Auernik et al., 2008; Liu et al., 2011b). *M. tengchongensis* Ric-A, the newest member of *Metallosphaera*, which was isolated from a sulfuric hot spring in Tengchong, Yunnan Province, China, showed an excellent performance in copper extraction from chalcopyrite (Peng et al., 2015). To better understand the unique metabolism, adaptation for extreme thermal and acidic conditions, roles in biogeochemical cycling, and the evolutionary history of the genus *Metallosphaera*, we performed genome sequencing of *M. tengchongensis* Ric-A and compared its genome with 18 available *Metallosphaera* genomes, of which 12 are from Genbank database of National Center for Biotechnology Information (NCBI) (*M. sedula* DSM5348, ARS50-1, ARS50-2, ARS120-1, ARS120-2, SARC-M1, and CuR1; *M. hakonensis* HO1-1 and JCM8857; *M. cuprina* Ar-4; *M. yellowstonensis* MK-1; *Metallosphaera* sp. UBA165) and six scaffold genomes are from the Integrated Microbial Genomes and Microbiomes (IMG/M) system of DOE Joint Genome Institute (JGI) (*Metallosphaera* spp. My-r02, My-r05, My-r06, YNP_08, YNP_14, and Obs4) (Table 1). In this work, we performed comprehensive analyses of genome-based phylogenetic relationships, metabolic pathway and gene function, heavy metal resistance, adhesion and motility, as well as mobile genetic elements (MGEs) and selective pressure. These findings will improve our understanding of the adaptive strategies of the organisms to their harsh environment and provide clues to design biomining or bioremediation processes in the future.

**TABLE 1:** The genomic statistics information and source of 19 strains used in this study.

Organism/name	Strain	Genbank or JGI accession number	Level and database	Size (Mb)	Coding density (%)	GC%	Gene	Protein	Environment	Address
*M. tengchongensis*	Ric-A	2821472399/CP049074	Complete NCBI	2.10	85.4	44.8	2331	2295	Acidic hot spring	China: Yunnan
*M. sedula*	DSM 5348	CP000682.1	Complete NCBI	2.19	89.3	46.2	2377	2298	Solfataric field	Italy
*M. sedula*	ARS50-1	CP012172.1	Complete NCBI	2.19	89.4	46.2	2375	2297	Lab	United States
*M. sedula*	ARS50-2	CP012173.1	Complete NCBI	2.19	89.4	46.2	2377	2298	Lab	United States
*M. sedula*	ARS120-1	CP012174.1	Complete NCBI	2.19	89.3	46.2	2376	2298	Lab	United States
*M. sedula*	ARS120-2	CP012175.1	Complete NCBI	2.19	89.3	46.2	2376	2298	Lab	United States
*M. sedula*	SARC-M1	CP012176.1	Complete NCBI	2.19	89.3	46.2	2379	2301	Lab	United States
*M. sedula*	CuR1	CP008822.1	Complete NCBI	2.19	89.2	46.2	2373	2289	Lab	United States
*M. hakonensis*	HO1-1	CP029287.1	Complete NCBI	2.54	80.3	43.7	2785	2736	Acidic hot Spring	Japan
*M. hakonensis*	JCM 8857	GCA_001315825.1	Draft NCBI	2.39	81.5	43.3	3312	3292	Acidic hot Spring	Japan
*M. cuprina*	Ar-4	CP002656.1	Complete NCBI	1.84	87.9	42.0	1968	1894	Acidic hot spring	China: Yunnan
*Metallosphaera* sp.	UBA165	GCA_001652185.1	Draft NCBI	1.83	88.5	45.7	3186	2821	Hot spring	Taiwan
*M. yellowstonensis*	MK1	GCA_000243315.1	Draft NCBI	2.82	82.4	47.7	3411	3356	Acidic hot spring	United States: Yellowstone National Park
*Metallosphaera* sp.	My-r02	2522125033	Draft IMG-M	1.85	82.5	47.4	2419	2366	Acidic hot spring	United States: Yellowstone National Park
*Metallosphaera* sp.	My-r05	2551306706	Draft IMG-M	1.85	87.9	47.4	1561	1520	Acidic hot spring	United States: Yellowstone National Park
*Metallosphaera* sp.	My-r06	2551306703	Draft IMG-M	1.85	89.0	48.3	1496	1455	Acidic hot spring	United States: Yellowstone National Park
*Metallosphaera* sp.	YNP_14	2502873002	Draft IMG-M	1.38	84.5	47.2	2093	2061	Thermal springs	United States: Yellowstone National Park
*Metallosphaera* sp.	YNP_08	2502894001	Draft IMG-M	1.10	78.4	43.9	2275	2252	Thermal springs	United States: Yellowstone National Park
*Metallosphaera* sp.	Obs4	2770939403	Draft IMG-M	2.70	83.2	50.4	3060	2968	Hot spring sediment	United States: Yellowstone National Park

## Materials and Methods

### Sample Collection and Sequencing

*Metallosphaera tengchongensis* strain Ric-A was isolated from the muddy water samples of sulfuric hot springs (24.57 N and 98.26 E, with the temperature range of 55–96°C and a pH range of 2.5–7.5, dissolved oxygen range of 0.01–1.00 mg/L) in Tengchong county of Yunnan Province, China. The hot springs are rich in S (SO_4_^2–^, 701.20–22.46 mg/L), Fe (13.89–0.05 mg/L), Ca (71.55–1.12 mg/L), K (22.64–63.01 mg/L), Al (42.43–0.13 mg/L), and other elements (Liu et al., 2011a; Peng et al., 2015; Qin et al., 2019). The sample was concentrated by tangential flow ultrafiltration through a hollow fiber membrane (Tianjin MOTIMO Membrane Technology, China). An aerobic enrichment culture in the flask with filtration membrane was established by inoculating the concentrate in basal salts medium (BSM) with elemental sulfur as energy source. The compositions of BSM were L^–1^: (NH_4_)_2_SO_4_, 3 g; K_2_HPO_4_ ⋅ 3H_2_O, 0.5 g; MgSO_4_ ⋅ 7H_2_O, 0.5g; KCl, 0.1 g; Ca(NO_3_)_2_, 0.01 g, added with 1 ml of trace element solution (FeCl_3_ ⋅ 6H_2_O, 1.1 g; CuSO_4_ ⋅ 5H_2_O, 0.05 g; H_3_BO_3_, 0.2 g; MnSO_4_ ⋅ H_2_O, 0.2 g; Na_2_MoO_4_ ⋅ 2H_2_O, 0.08 g; CoCl_2_ ⋅ 6H_2_O, 0.06 g; ZnSO_4_ ⋅ 7H_2_O, 0.09 g in 1 L of distilled water). After static culture for 5–7 days at 65°C, samples of the grown culture were spread on BSM solid plates with potassium tetrathionate (K_2_S_4_O_6_, 10 mmol/L) or yeast extract (1 g/L) as energy source. The plates were incubated for 7 days at 65°C. Colonies were picked and purified by re-plating. The purified strain Ric-A was grown at 70°C in BSM (pH 2.0) supplemented with 1 g/L yeast extract. The stationary-phase cells were harvested by centrifugation. The genomic DNA was extracted from the concentrated cells according to the instruction of “JGI Bacterial DNA isolation CTAB-2012.”^1^ After checking its quality, DNA was fragmented and the fragments were end-repaired and polyadenylated, and then ligated to sequencing adapter. SMRTbell DNA library was constructed by using Blue Pippin Size-Selection System; library quality was evaluated by Qubit 3.0 Fluorometer (Life Technologies, Grand Island, NY, United States) and sequenced by PacBio Biosciences (PacBio) RSII and Genome Analyzer IIx sequence platforms at Chinese National Human Genome Center at Shanghai (CHGC). After sequencing, the low-quality reads were filtered by the SMRT 2.3.0 (Chin et al., 2013), and the filtered reads were assembled to generate one contig without gaps. The hierarchical genome-assembly process (HGAP.3) pipeline implemented in SMRT Analysis 2.3.0^2^ was used to correct for random errors in the long seed reads (seed length threshold 6 Kb) by aligning shorter reads from the same library against them. The resulting corrected, preassembled reads were used for *de novo* assembly. Genome data of 18 previously sequenced strains belonging to *Metallosphaera* were collected from NCBI and IMG-M database. The detailed genomic statistics information and source of 19 *Metallosphaera* strains used in this study were summarized in Table 1.

### Average Nucleotide Identity and Whole Genome Alignments

Comparisons of average nucleotide identity (ANI) based on Blast algorithm were conducted using the pyani module^3^ with default parameters. We applied the “progressive Mauve program” within Mauve v 2.3.0 (Darling et al., 2004) for constructing and visualizing multiple genome alignments of *M. tengchongensis* Ric-A with four other available complete genome sequences of *M. sedula* DSM 5348, *M. hakonensis* HO1-1, *M. cuprina* Ar-4, and *M. yellowstonensis* MK1. BlastN-based whole-genome comparison of strains *M. cuprina* Ar-4, *M. hakonensis* HO1-1, *Metallosphaera* sp. My-r06, *M. sedula* DSM 5348, *M. tengchongensis* Ric-A, and *M. yellowstonensis* MK1 were performed and represented with BRIG-0.95 (Alikhan et al., 2011), and these strains were used as references, respectively. GC content and GC skew of each genome were also indicated.

### Phylogenomic Analyses

We constructed a phylogenetic tree of the 19 *Metallosphaera* spp. genomes based on whole-genome sequences with CVTree3 (Xu and Hao, 2009). The phylogenetic tree of the 19 genomes based on concatenation of the 85 core genes in a genome was constructed with the neighbor-joining (NJ), UPGMA, and maximum-likelihood (ML) method using MEGA-X (Kumar et al., 2018) with 1000 bootstrap replicates.

### Genome Annotation

We applied Prokka (Seemann, 2014) and IMG Annotation Pipeline v.4.16.0 (Chen et al., 2017) for genome annotation and putative horizontally transferred gene detection. We performed Diamond BlastP v0.9.24 (Buchfink et al., 2015) with a cutoff e-value of 1e^–5^ together with the dbCAN database v2.0 (Le et al., 2018) for identification of genes related to carbohydrate activity enzyme (CAZymes). The BacMet v2.0 database (Pal et al., 2014) that contained genes with experimentally confirmed metal resistance function was used to identify the genes associated with metal resistance in *Metallosphaera* genomes. Gene annotations based on COG (Tatusov et al., 2001), Pfam (Finn et al., 2014), and TIGRFAM (Selengut et al., 2007) databases were performed via WebMGA (Wu et al., 2011) using Blast with a cutoff e-value of 1e^–5^.

### Prediction of Mobile Genetic Elements

We applied the ISFinder (Siguier et al., 2006)^4^ to predict and classify insertion sequences (IS) and transposases within *Metallosphaera* genomes with Blastp v0.9.24 (cutoff e-value of 1e^–5^). We applied the IslandViewer 4 (Bertelli et al., 2017),^5^ which integrated prediction methods including IslandPath-DIMOB and SIGI-HMM that analyzed sequence composition, and another comparative genomic islands (GIs) prediction method IslandPick, to detect putative GIs distributed over *Metallosphaera* genomes. We applied PHASTER (Phage Search Tool Enhanced Release) (Arndt et al., 2016)^6^ for detection and annotation of prophage and prophage remnant sequences within *Metallosphaera* genomes. We also applied CrisprCasFinder (Couvin et al., 2018)^7^ for detection of CRISPRs and Cas genes within *Metallosphaera* genomes.

### Comparative Genomic Analyses of Metallosphaera

The Bacterial Pan Genome Analyses tool (BPGA) pipeline (Chaudhari et al., 2016) was used to perform pan/core-genome analyses and calculation applying default parameters. The size of the *Metallosphaera* pan-genome was fitted into a power law regression function *P*_*s*_ = κ*n*^γ^ with a built-in program of BPGA (Chaudhari et al., 2016), in which *P*_*s*_ was the total number of gene families, *n* stood for the number of tested genomes, and γ was free parameters. If exponent γ < 0, then the pan-genome of *Metallosphaera* was suggested to be “closed.” In this case, the size of the pan-genome is relatively constant, even if new genomes were added into the analysis. On the contrary, the pan-genome was suggested to be “open” in the case of 0 < γ < 1. In addition, the size of the core-genome of *Metallosphaera* was fitted into an exponential decay function *F*_*c*_ = κ*_*c*_*exp(−*n*/*τ_*c*_*) with a built-in program of BPGA pipeline (Chaudhari et al., 2016), in which *F*_*c*_ stood for the number of core gene families, while κ*_*c*_* and *τ_*c*_* were free parameters. Gene family clustering followed by genome-wide comparisons of five *Metallosphaera-*type strains including *M. tengchongensis* Ric-A, *M. sedula* DSM 5348, *M. hakonensis* HO1-1, *M. cuprina* Ar-4, and *M. yellowstonensis* MK1 together with UniProt search, Gene Ontology (GO) Slim annotation, and GO enrichment analyses (default cutoff *p*-value is 0.05) were performed via OrthoVenn (Wang et al., 2015)^8^ with default parameters.

### Gene Family Evolution Analyses

Count is a software designed to analyze numerical profiles of homologous gene families on a phylogeny, which can execute ancestral reconstructions and predict family- and lineage-specific characteristics along the evolutionary tree (Miklós, 2010). We used Count software, combined BPGA v2.0 pipeline, and Wagner parsimony algorithm (Farris, 1970) for gene family clustering, ancestor genome size estimation, and detecting gene family gain and loss events, together with family expansion and contraction events with penalty ratio set to 1. We conducted the analyses only on five *Metallosphaera-*type strains, including *M. tengchongensis* Ric-A, *M. sedula* DSM 5348, *M. hakonensis* HO1-1, *M. cuprina* Ar-4, and *M. yellowstonensis* MK1 taken into consideration, due to the estimation requiring complete sets of testing gene families available only in species with high-quality genome databases.

### Selective Pressure Analyses

We detected the numbers of sites under negative (purifying) or positive (diversifying) selection and estimated global dN/dS values of each gene family that contained more than three non-identical sequences (due to limitation of HyPhy) based on multiple alignments of orthologous codon sequences and a tree topology by means of HyPhy package (Pond and Muse, 2005) using the Fixed Effects Likelihood (FEL) method (Kosakovsky Pond and Frost, 2005) (applied a likelihood ratio test with default cutoff *p*-value: 0.1) via datamonkey server (Weaver et al., 2018).^9^ The coding sequences of five *Metallosphaera-*type strains, including *M. tengchongensis* Ric-A, *M. sedula* DSM 5348, *M. hakonensis* HO1-1, *M. cuprina* Ar-4, and *M. yellowstonensis* MK1, were aligned with muscle codon alignment module implemented in MEGACC (Kumar et al., 2012) to obtain final codon alignments.

## Results and Discussion

### General Genome Features of *M. tengchongensis* and Other Strains

**FIGURE 1:**
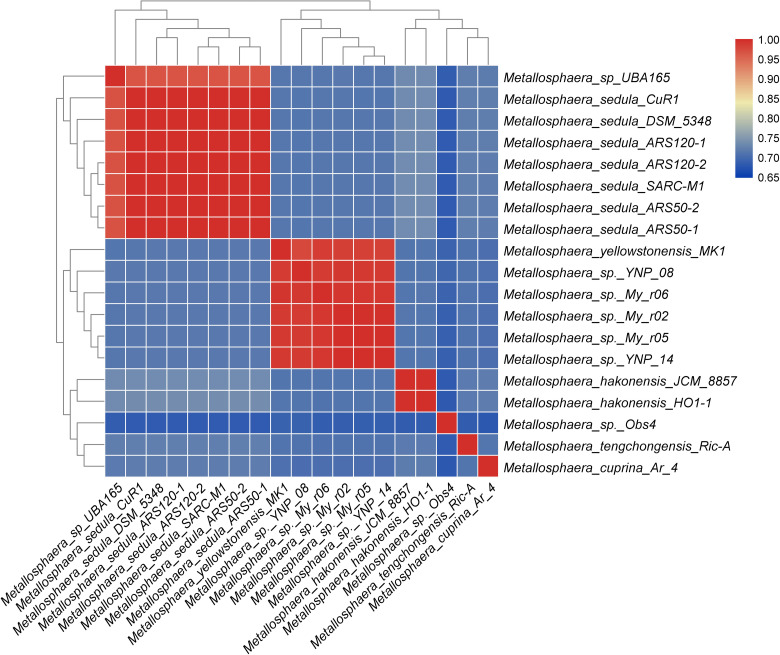
Heat map showing species boundary of genus *Metallosphaera* based on ANI value (cutoff value = 96%), which is a universally accepted genomic measure for prokaryotic species delineation (Richter and Rossello-Mora, 2009).

A total of 2331 CDS, including 32 tRNA and 4 rRNA, were predicted in the genome of *M. tengchongensis* strain Ric-A using Prokka (Seemann, 2014). Whole-genome BLASTN-based ANI analyses showed only an ANI of 72.3% compared with the closest *Metallosphaera* genome (Supplementary Table S1). A summary of features for the 19 *Metallosphaera* genomes is listed in Table 1. *M. yellowstonensis* MK1 possessed the largest genome (2.82 Mb). The G + C contents of the 19 genomes ranged from 42.0 to 50.4%. These genomes varied in coding density from 78.4 to 89.4%, indicating substantial intra-genus differences. We determined that the previously unclassified strain UBA165 was a member of *M. sedula*, and strains My-r02, My-r05, My-r06, YNP_08, and YNP_14 were members of *M. yellowstonensis* based on an ANI cutoff of 96% (Richter and Rossello-Mora, 2009), as supported by further phylogenetic analyses (Figure 1, Supplementary Figure S1, and Supplementary Table S1). The genome alignment of *M. tengchongensis* Ric-A with four other complete genomes of *Metallosphaera* strains using Mauve (Darling et al., 2004) indicated that the chromosomal alignments of *Metallosphaera* genomes were non-conserved, as shown by the presence of hundreds of poorly organized collinear blocks and numerous inversed and rearranged regions (Supplementary Figure S2). Each *Metallosphaera* species harbored genomic regions that were not commonly shared; most of these harbored poorly characterized proteins as revealed by whole genome comparison of *Metallosphaera* spp. using BRIG (Alikhan et al., 2011) (Supplementary Figures S2, S3).

### Core- and Pan-Genome and Phylogenomic Analysis of Metallosphaera

**FIGURE 2:**
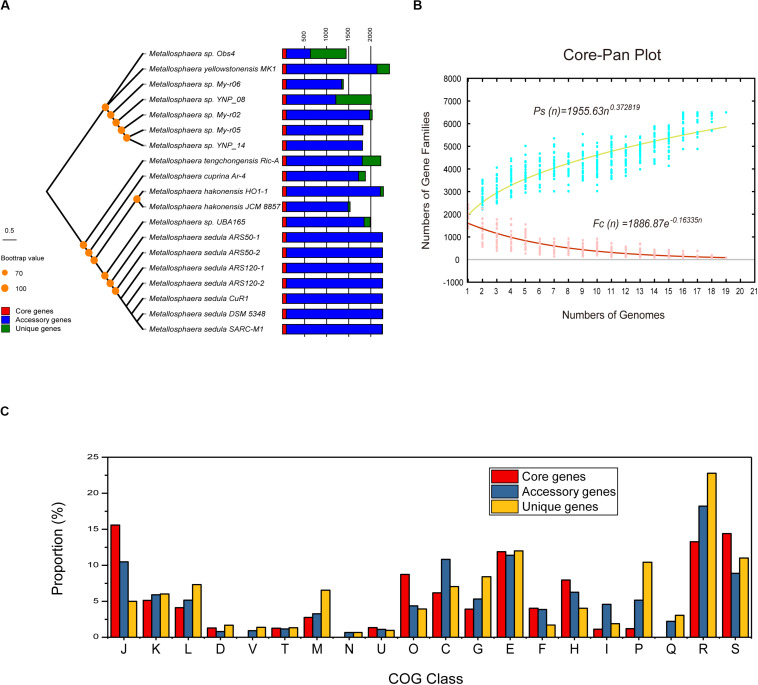
Pan-genome analyses of strains in the genus *Metallosphaera*. **(A)** NJ phylogenetic tree based on concatenated 85 core genes (1000 bootstrap replicates) of 19 *Metallosphaera* strains (left) and stack bar diagram showing sizes of orthologous genes shared by all strains (i.e., the core-genome), number of orthologous genes shared by partial strains (i.e., the accessory genome), and number of strain-specific gene families (i.e., the unique gene) in each strain. **(B)** The curve of the fitting function and the estimation of pan-genome and core-genome sizes of *Metallosphaera. Ps* and *F*_*c*_ stand for the total number of gene families and the number of core gene families, respectively. More details for modeling approaches are presented in Section 2.6 “Comparative Genomic Analyses of *Metallosphaera*”. **(C)** Bar chart showing proportions of COG categories of the different parts of *Metallosphaera* pan-genome (i.e., core, accessory, unique). COG categories description (J, translation, ribosomal structure and biogenesis; K, transcription; L, replication, recombination and repair; D, cell cycle control, cell division, chromosome partitioning; T, signal transduction mechanisms; M, cell wall/membrane/envelope biogenesis; N, Cell motility; U, intracellular trafficking, secretion, and vesicular transport; O, posttranslational modification, protein turnover, chaperones; C, energy production and conversion; G, carbohydrate transport and metabolism; E, amino acid transport and metabolism; F, nucleotide transport and metabolism; H, coenzyme transport and metabolism; I, lipid transport and metabolism; P, inorganic ion transport and metabolism; Q, secondary metabolites biosynthesis, transport and catabolism; R, general function prediction only; S, function unknown).

The phylogenetic trees based on the concatenated alignment of 85 core genes inferred with NJ, ML, MP, and UPGMA methods were congruent with each other, and the phylogenetic tree based on whole-genome analyses was congruent with the core-gene phylogenetic tree (Supplementary Figures S4, S5). Strain Obs4 is located on a clade apart from other *Metallosphaera* strains (Supplementary Figures S1, S4, S5). However, strain Obs4 should still be considered a member of genus *Metallosphaera* based on percentage of conserved proteins (POCP). Strain Obs4 had a POCP of 50.2% against *M. yellowstonensis* MK1, within the genus cutoff value of 50% (Qin et al., 2014). The pan-genome of 19 *Metallosphaera* strains possessed 6499 gene families, while the core-genome possessed only 85 gene families. Core- and pan-genome analyses of the 19 *Metallosphaera* genomes revealed an “open” pan-genome fitted into a power law regression function [*P*_*s*_ (*n*) = 1955.63 *n*^0.372819^] with a parameter (γ) of 0.372819 falling into the range 0 < γ < 1. The core-genome was fitted into an exponential regression [*F*_*c*_ (*n*) = 1886.87 *e*^–0.16335^ *^*n*^*], which had a steep slope, reaching a minimum of 85 gene families after the 19th genome was added (Figure 2B). The result of COG annotation revealed that the core-genome had a higher proportion of genes involved in COG categories that are associated with central biological functions translation, ribosomal structure, and biogenesis (J); posttranslational modification, protein turnover, chaperones (O); and coenzyme transport and metabolism (H) than the accessory genome and strain-unique genome. In contrast, the accessory genome had a higher proportion of genes related to COG energy production and conversion (C) and lipid transport and metabolism (I). We found that strain-specific gene families had a higher proportion of genes categorized in COG replication, recombination and repair (L), cell wall/membrane/envelope biogenesis (M), inorganic ion transport and metabolism (P), and carbohydrate transport and metabolism (G) (Figure 2). We propose that these genes are associated with adaptive evolution within the genus *Metallosphaera*. The oligotrophic, metal-laden, and extremely acidic environments select for a highly efficient DNA injury repair system and a flexible trophic mode in *Metallosphaera*. The genomic diversity and specificity of different *Metallosphaera* strains are reflecting their distinct survival strategies in different environments.

### Metabolic and Functional Potential

#### Sulfur Metabolism

**FIGURE 3:**
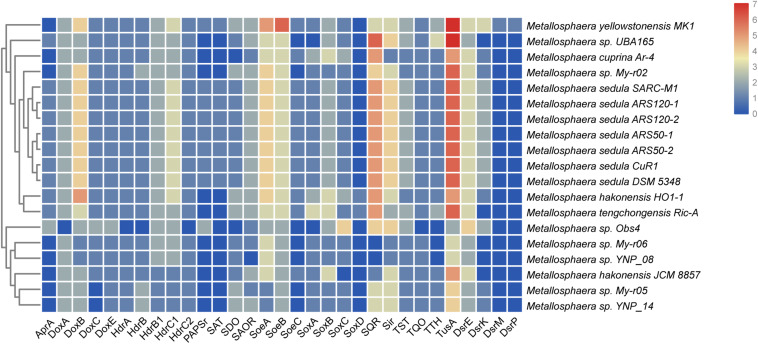
Heat map showing the distribution and numbers of genes encoding putative proteins in sulfur metabolism in various *Metallosphaera* strains. *AprA*, APS reductase; *SAT*, ATP sulfurylase; *TST*, thiosulfate sulfurtransferase; *SDO*, sulfur dioxygenase; *DoxA/DoxD*, thiosulfate:quinone oxidoreductase subunits; *Sir*, sulfite reductase; *TTH*, tetrathionate hydrolase; *HdrA/HdrB/HdrC*, heterodisulfide reductase complex subunits; *PAPSr*, phosphoadenosine phosphosulfate reductase; *SQR*, sulfide:quinone oxidoreductase; *SoeABC*, sulfite-oxidizing enzyme; *CbsAB/SoxNL*, terminal oxidase complex; *DoxE*, sulfocyanin; *MCO*, multicopper blue protein; *doxBCE*, terminal oxidase complex; *TusA/DsrE*, cytoplasmic sulfur trafficking proteins.

Sulfur and hydrogen are important in energy flow in thermal environments, such as marine hydrothermal systems, continental solfataras, and hot springs, where many bacteria and archaea can grow by oxidizing hydrogen, sulfide, elemental sulfur, and thiosulfate (Amend and Shock, 2001; Zeldes et al., 2019). It was reported that *Metallosphaera* could utilize different sulfur compounds as energy sources for growth (Peng et al., 2015). The genes *sqr* encoding for sulfide:quinone oxidoreductase (SQR), which catalyzes the oxidation of hydrogen sulfide forming polysulfide, were detected in all 19 genomes of *Metallosphaera*. Unlike the genus *Acidianus* and certain members of *Sulfolobus*, the genes encoding the homologs of sulfur oxygenase/reductase (SOR), a key enzyme for archaeal sulfur oxidation (Kletzin, 1992; Chen et al., 2005; Urich et al., 2006; Dai et al., 2016), were not found in the genomes of all strains of *Metallosphaera*. However, sulfur dioxygenases (SDO, *sdo*) were encoded in all strains except for *M. cuprina* and *Metallosphaera* sp. Obs4 (Figure 3). In addition, sulfite-acceptor oxidoreductases (SAOR, *saor*) genes were also detected in all strains (Figure 3). *Metallosphaera* spp. also harbored genes encoding for tetrathionate hydrolase (TTH, *tth*), sulfite reductase (SIR, *sir*), and genomic clusters encoding for the thiosulfate:quinone oxidoreductase (TQO) subunits (*doxA/doxD*) and heterodisulfide reductase (HDR, *hdrC1-hdrB1A-hyp-hdrC2-hdrB2*) complex. The genes encoding for thiosulfate sulfurtransferase (TST, *tst*) were also detected in all species (Figure 3). According to the genome composition, we speculate that the pathway of sulfur metabolism for *Metallosphaera* might be SDO oxidized sulfur to sulfite, SAOR oxidized sulfite to sulfate, and TQO was responsible for the transformation of thiosulfate to tetrathionate, whereas TTH catalyzed the tetrathionate hydrolysis into thiosulfate, sulfur, and sulfate. However, the function of some proteins, especially for SDO and SAOR, had not been verified in *Metallosphaera* members. TST and HDR catalyzed the mutual conversion of thiol proteins (RSH) and sulfane sulfates (RSSH) in the cytoplasm (Chen et al., 2012), and HDR was also implicated in transferring electrons to reduce Fd_ox_ (Hua et al., 2018). Besides, *tusA-dsrE2-dsrE3A* gene clusters that functioned in cytoplasmic sulfur trafficking and dissimilatory tetrathionate oxidation were found located next to the *hdr* clusters in all *Metallosphaera* genomes, similar to other Sulfolobales members (Urbieta et al., 2017). The subunits SoeAB of the heterotrimeric membrane-bound sulfite-oxidizing enzyme complex SoeABC were detected in all *Metallosphaera* genomes, but the subunit SoeC was not identified in *Metallosphaera* sp. UBA165, *Metallosphaera* sp. Obs4, and other strains of My-r02, YNP-08, My-r06, My-r05, and YNP-14, probably resulting from the incompleteness of these genomes. Genes coding ATP sulfurylase (SAT, *sat*), adenosine-5′-phosphosulfate reductase (APR, *apr*) subunit AprA, and phosphoadenosine phosphosulfate reductase (PAPSr, *papsr*) that are involved in adenosine-5′-phosphosulfate (APS) pathway were only detected in *M. sedula* and *M. yellowstonensis* (Figure 3). Genes encoding for *soxABC* and *doxBCE* complex involved in sulfur oxidation and electron transfer were also annotated in *Metallosphaera* genomes. It was reported that sulfite was readily oxidized to sulfate through the direct SoeABC pathway and/or the indirect APS pathway presented in the cytoplasm in the purple sulfur bacterium *Allochromatium vinosum* (Christiane et al., 2013). Whether these pathways work in other phototrophic sulfur bacteria and sulfur-oxidized archaea remains unknown. Genes (e.g., *hyn*S, *hyn*L, *hox*M, *hyp*C, *hyp*D, and *isp* 1) encoded for the structural and auxiliary proteins of Ni/Fe hydrogenase that are potentially associated with electron transfer between hydrogen and sulfur-containing compounds were only detected in *M. sedula* and *M. yellowstonensis*; however, genes *hyp*E, *hyn*Y, and *isp* 2 were presented in *all* analysis strains. No genes of h*yp*Z, *hyn*Z, and *hyp*Y were found on the genomes of all strains (Laska et al., 2003) (Supplementary Figure S6).

#### Iron Metabolism

**FIGURE 4:**
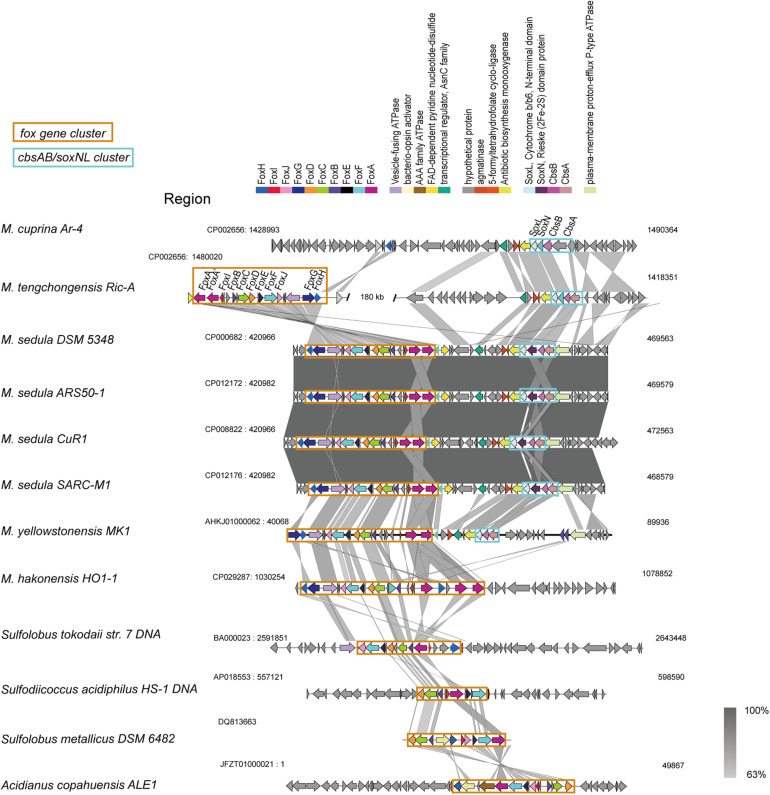
Synteny analysis of *fox* gene clusters and *soxNL/cbsAB* gene clusters derived from representative *Metallosphaera* strains and other representative species in *Sulfolobales*.

The *fox* genes involved in iron oxidation (Kozubal et al., 2011) were detected in all *Metallosphaera*-type species, but *M. cuprina* may have lost the subunits *foxD*, *foxE*, *foxF*, and *foxI* (Supplementary Figure S7). The arrangement of *fox* genes was similar in *Metallosphaera* spp. following the pattern *foxA-A*′*-I-B-C-D-E-F-J-G-H*, but it is different from other members of iron-oxidizing Sulfolobales (Figure 4), and in *M. hakonensis* HO1-1, a third copy of *foxA* was found within this cluster, probably resulting from gene duplication (Figure 4). The transcription initiation directions of these *fox* genes were not consistent, and their open reading frames (ORFs) were separated by spacers (Figure 4). Genes encoding for terminal oxidase complex *cbsAB/soxNL* were found downstream of *fox* cluster in *M. sedula* and *M. yellowstonensis*, while in strain *M. tengchongensis* Ric-A, these two clusters were separated by a genome region of about 180 kb (Figure 4). A gene for sulfocyanin (SoxE), a blue copper-containing protein that may function as a temporary electron storage or electron carrier in the iron-oxidizing electron transport chain (Kozubal et al., 2011), occurred in all *Metallosphaera* genomes in this study. Genes encoding for multicopper blue protein (*mco*) that contained two plastocyanin type I copper domains were only detected in *M. yellowstonensis* and *M. sedula* (Kozubal et al., 2011). These oxidases may couple the reduction of oxygen to proton translocation in cooperation with Fox complex mentioned-above (Auernik and Kelly, 2008; Kozubal et al., 2011).

#### Carbon Metabolism

*Metallosphaera* spp. contained an abundant repertoire of carbohydrate-active enzymes (CAZymes) including carbohydrate esterases (CEs), carbohydrate binding molecules (CBMs), glycosyltransferases (GTs), glycoside hydrolases (GHs), auxiliary activity proteins (AAs), and a small number of polysaccharide lyases (PLs), of which GTs were most abundant, and *Metallosphaera* sp. Obs4 possessed the most carbohydrate active enzymes (Supplementary Figure S7). The diversity of encoding genes for CAZymes possibly reflected different carbohydrate metabolism strategies in *Metallosphaera*. A complete set of genes encoding for glycolysis, gluconeogenesis, the archaeal pentose phosphate pathway, as well as an atypical TCA cycle (replacing genes encoding the alpha-ketoglutarate dehydrogenase with those encoding 2-oxoacid:ferredoxin oxidoreductase) were detected in all *Metallosphaera* species. *Metallosphaera* spp. also contained complete non-phosphorylative and semi-phosphorylative Entner–Doudoroff (ED) pathways. Genes encoding homologous enzymes for the complete 3-hydroxypropionate/4-hydroxybutyrate (HP/HB) cycle and the dicarboxylate/4-hydroxybutyrate (DC/HB) cycle involved in autotrophic carbon fixation (Berg et al., 2007, 2010; Jiang et al., 2014) were also found in all *Metallosphaera* genomes (Supplementary Figure S9). It is reported that carbon monoxide was ubiquitous in hydrothermal habitats (King and Weber, 2007). All members of *Metallosphaera* in this study possessed putative type I carbon monoxide dehydrogenase (CODH) encoded by gene cluster *coxLSM*; however, the typical active site motif (VAYRCSFR) of CODH (Dobbek et al., 2002) was not observed in these proteins, which indicated that these CODH probably do not use CO; alternatively, they could possess a novel active site motif (Supplementary Figure S9).

#### Nitrogen Metabolism

All *Metallosphaera* strains possessed genes encoding for nitrate reductase and nitrite reductase involved in assimilatory nitrate reduction. Except for *nar*G genes in seven strains of *M. sedula*, the *nar*GHJI operon encoding for dissimilatory nitrate reductase was only found in *M. yellowstonensis* MK1 (Supplementary Figure S10). This operon was located on a GI in *M. yellowstonensis* MK1, suggesting that it might be acquired through horizontal gene transfer (HGT) events (Supplementary Table S2; Bertelli et al., 2017). Urease gene clusters consisting of the functional subunits (*ureAB* and *ureC*) and accessory proteins (*ureE*, *ureF*, and *ureG*) were found in all Metallosphaera species except for *M. yellowstonensis* (Supplementary Figure S10), indicating that urea assimilation was employed by most *Metallosphaera* strains to provide sufficient ammonia by converting one urea molecule into one carbon dioxide molecule plus two ammonia molecules. Genes encoding for nitrilase and formamidase, metabolizing organic nitrogen to ammonia, and genes encoding for ammonium transport (*amt*) and ammonia-dependent biosyntheses, such as carbamoyl-phosphate synthases (*carAB*), glutamate dehydrogenases (*gdhA*), and glutamine synthetases (*glnA*), were found in the genomes of all *Metallosphaera* species (Supplementary Figure S10). There is also a complete set of genes involved in arginine synthesis in all Metallosphaera species (Supplementary Figure S10). Polyamine derived from arginine can stabilize DNA by protecting DNA from free radical attacks and thermal denaturation (Abby et al., 2018). Polyamine biosynthesis-related genes that encoded for agmatinase (*speB*), S-adenosylmethionine decarboxylase (*speD*), and spermidine synthase (*speE*) were found in the genomes of all Metallosphaera species, while the arginine decarboxylase gene (*speA*) for the first two steps of putrescine biosynthesis was not detected; its function may have been substituted by S-adenosylmethionine decarboxylase (*speD*) (Giles and Graham, 2008).

#### Heavy Metal Resistance

*Metallosphaera* can survive natural and anthropogenic metal-rich environments, and BacMet database annotations revealed an abundant repertoire of heavy metal resistance genes in *Metallosphaera* spp., exhibiting diverse strategies to avert the deleterious effect of toxic metals on biological function (Supplementary Figure S11). Most of the genes related to arsenic resistance (*aioAB*: arsenite oxidase; *arsABR*: arsenical pump-driving ATPase; *arsM*: methyltransferase), divalent-cation resistance (*copARZ*, *cueA*, *cutA*, *czcD*, *corRC*, *nccN*, *nikABCDER*, and *mntRH*), mercury resistance (*merA*: mercuric reductase), and iron regulation (*fecDE*, *furA*, *fbpC*) were found in all tested *Metallosphaera* genomes. Alkylmercury lyase (encoded by gene *merB*) that cleaved mercury-alkyl bonds for mercury detoxification (Melnick and Gerard, 2007) was only detected in *M. yellowstonensis.* Genes encoding for “DNA-binding protein from starved cells” (Dps) (Blake et al., 2005), which can physically shield DNA against oxidative damage, were only detected in strains of *M. sedula* and *Metallosphaera* sp. UBA1654. Dps proteins were also able to control Fenton reaction through storing ferric oxide as a mineral core on their interior cage surface (Blake et al., 2005).

#### Adhesion and Motility

Etracellular polysaccharides (EPS) play a significant role in cell adhesion and biofilm formation, which is closely related to colonization, mineral solubilizing ability, and protection against adverse environmental conditions (Basak et al., 2014; Marino et al., 2018; Yu et al., 2019). Gene clusters encoding for d-TDP-glucose pyrophosphorylase (*rfbA*), d-TDP-glucose 4,6-dehydratase (*rfbB*), d-TDP-4-dehydrorhamnose 3,5-epimerase (*rfbC*), and d-TDP-4-dehydrorhamnose reductase (*rfbD*), which can convert glucose-1-phosphate to the EPS precursor d-TDP-rhamnose via a series of reactions, were found in all Metallosphaera genomes (Supplementary Figure S9). Motility conferred by flagella can provide a competitive advantage for microorganisms to move toward beneficial conditions. From the genomic and functional analysis, it is found that the sequenced archaea maintain a unique flagellum composition and mode of assembly, distinct from the bacteria. Archaeal flagellin commonly anchors on a *fla* locus, which was encoded by 7–13 flagella-related genes. Studies of the flagella system of the crenarchaeal model strain *Sulfolobus acidocaldarius* identified seven *fla* genes called *fla*B, *fla*X, *fla*G, *fla*F, *fla*H, *fla*I, and *fla*J, and confirmed that all seven genes were essential for assembly and motility (Thomas et al., 2001; Lassak et al., 2012; Dominik et al., 2016). However, in the genomes of 19 strains of *Metallosphaera*, genes encoding for crenarchaeal flagellin (*flaB*) and flagella accessory proteins (*flaH*, *flaI*, *flaG*, *flaF*, and *flaJ*) were only detected in strains of *M. sedula*, *M. yellowstonensis*, and *Metallosphaera* sp. UBA165. Further, a unique crenarchaeal gene *flaX*, coding for a structural part of the archaeal flagellum assembly apparatus only present in *Metallosphaera* sp. Obs4 and *Metallosphaera* sp. My-r06 (Supplementary Figure S12). The roles of FlaX and other accessory proteins in *Metallosphaera* members need further experimental verification. Genome comparative results indicated that the flagellum assembly apparatus did not present in every genome of these strains, which is consistent with the motility difference of *Metallosphaera* strains known by their physiological research (Peng et al., 2015). The genomes of *M. tengchongensis* Ric-A and *M. hakonensis* DSM 7519 only contain Fla L. Both species do not possess flagella and do not have motility (Peng et al., 2015).

### Mobile Genetic Elements and CRISPR-Cas Systems

Mobile genetic elements, including GIs, IS, transposons, and phages, are genome segments that display intra- and/or extracellular translocation abilities associated with HGT (Springael and Top, 2004). The prokaryotic CRISPR (clustered, regularly, interspaced, short, palindromic repeats)-Cas (CRISPR-associated genes) systems are attested to confer resistance to viral attack and mediate interactions between the host and phage. The results showed that in *Metallosphaera*, the number of transposon sequences per genome ranged from 65 (*M. cuprina* Ar-4) to 283 (*M. yellowstonensis* MK1) and the number of GI-related sequences per genome ranged from 21 (*M. cuprina* Ar-4) to 487 (*M. yellowstonensis* MK1) (Supplementary Tables S2, S3). GI regions of *Metallosphaera* spp. harbored genes related to the formation of extracellular polysaccharides (EPS) that account for ∼2.4% of all sequences, defense and DNA repair systems (∼2.7%), and stress resistance (∼2.0%). These additional functionalities endowed by HGT events may facilitate adaptive survival and protect *Metallosphaera* against DNA damage and protein denaturation in its acidic hot spring habitat (Yu et al., 2019). The number of prophages and prophage remnants ranged from 0 to 27 (*M. sedula* DSM5348) (Supplementary Table S3). Type I-A and/or type III-A/D CRISPR-Cas systems were found in all the genomes in this study except for *Metallosphaera* sp. My-r06. *M. tengchongensis* Ric-A contained the most (410) CRISPR-Cas-related genes or spacers. CRISPR-Cas-related sequences were detected in predicted GI regions of all *Metallosphaera* spp. except for *M. cuprina* Ar-4, reflecting the mobility of CRISPR-Cas systems (Koonin and Makarova, 2017; Krupovic et al., 2017; Peters et al., 2017).

### Gene Ontology Enrichment and Evolutionary Analyses of Five *Metallosphaera*-Type Strains

**FIGURE 5:**
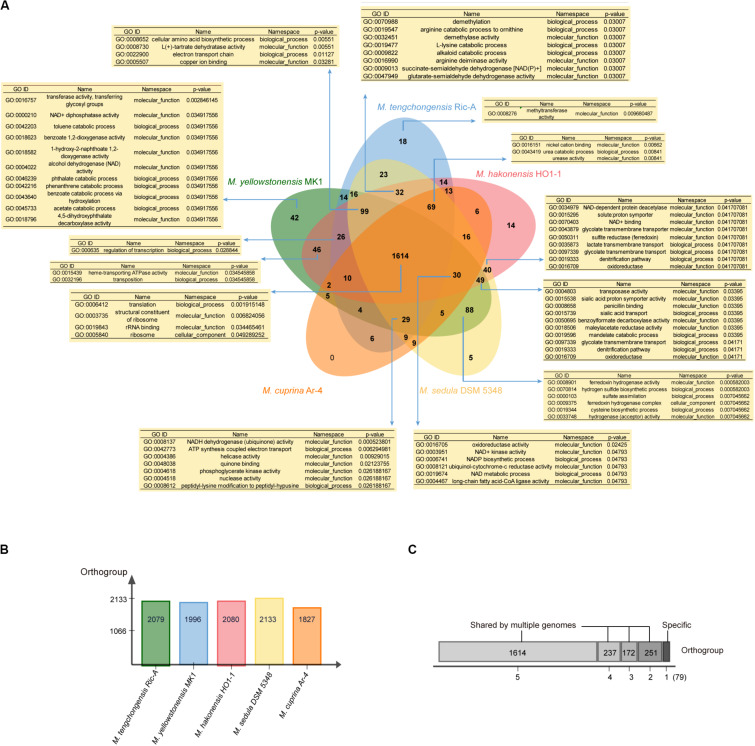
**(A)** Venn diagram showing the numbers of orthogroup including the core-genome (shared by all five *Metallosphaera* type strains), the accessory genome (shared by some but not at all of these strains), and strain-specific gene families in individual genomes; enriched Gene ontology (GO) terms (*p* < 0.05) in respective orthogroups are shown in the tables. **(B)** Bar chart showing the total numbers of orthogroup identified in each genome of the five *Metallosphaera*-type strains. **(C)** The total numbers of orthogroups of strain-specific gene families (=1) or the accessory genome (shared by 2, 3, and 4 strains) and core-genome of five *Metallosphaera*-type strains.

We further applied the software OrthoVenn (Yi et al., 2015) and Count (Miklós, 2010) for gene clustering, GO enrichment analyses, and gene family evolutionary analyses of five *Metallosphaera-*type strains including *M. tengchongensis* Ric-A, *M. sedula* DSM 5348, *M. hakonensis* HO1-1, *M. cuprina* Ar-4, and *M. yellowstonensis* MK1. Results showed that 1614 (68.6%) out of 2353 identified gene families were shared by all species. *M. sedula* DSM 5348 had the most gene families (2133) in its genome, whereas *M. cuprina* Ar-4 had the fewest (1827), and *M. yellowstonensis* MK1 possessed the most strain-specific gene families (42) followed by *M. tengchongensis* Ric-A (18) (Figure 5). GO enrichment analyses showed that in significantly enriched (*p*-value < 0.05) commonly shared gene families, GO terms were associated with basic biological functions including translation (GO:0006412), rRNA binding (GO:0019843), and ribosome-related function (GO:0005840; GO:0003735). Functions significantly enriched (*p*-value < 0.05) in accessory genes families were related to electron transport chain (GO:0022900; GO:0008137; GO:0042773; GO:0048038; GO:0006741; GO:0008121; GO:0003951), lipid metabolism-related long-chain fatty acid-CoA ligase activity (GO:0004467), and nutrient transport and catabolic process of nitrogen sources such as urea (GO:0043419; GO:0009039) and carbon sources such as lactate (GO:0035873), mandelate (GO:0019596), glycolate (GO:0097339), benzoylformate (GO:0050695), and maleylacetate (GO:0018506) related to heterotrophic lifestyle. Proton symporter activity (GO:0015295; GO:0015538) involved in pH homeostasis, amino acid biosynthesis-related denitrification pathway (GO:0019333), and sulfur metabolism-related functions including sulfate assimilation (GO:0000103), hydrogen sulfide biosynthetic process (GO:0070814), SIR (GO:0050311) together with hydrogenase-related functions (GO:0033748; GO:0008901), arginine metabolism (GO:0016990; GO:0019547), and cysteine biosynthesis process (GO:0019344) were also enriched (*p*-value < 0.05) in accessory gene families (Figure 5). These functions and pathways probably reflected the adaptation of *Metallosphaera* spp. to acidic, sulfur-rich, heat, and metal-laden environments. *Metallosphaera* may take advantage of hydrogen sulfide for cysteine biosynthesis. Disulfide bonds in thermophilic proteins are omnipresent in thermophiles and help stabilize proteins against the harsh conditions (Beeby et al., 2005). In addition, cysteine-rich proteins such as disulfide oxidoreductase can be used by microorganisms to chelate heavy metal ions in the cytoplasm so as to reduce metal-induced reactive oxygen species (ROS), which is supported by previous results that Cu^2+^ exposure induced assimilatory sulfur metabolism for cysteine biosynthesis in *Metallosphaera* (Wheaton et al., 2016; Meslé et al., 2017). Interestingly, functions enriched (*p*-value < 0.05) in strain-specific gene families of *M. yellowstonensis* MK1 were related to aromatic compound metabolism such as catabolic process of benzoate (GO:0018623; GO:0043640), phthalate (GO:0046239; GO:0018796), naphthoate (GO:0018582), toluene (GO:0042203), and phenanthrene (GO:0042216) (Figure 5), which suggested that *M. yellowstonensis* MK1 was apt at utilizing a broader spectrum of organic carbon sources possessing great potential in bioremediation. Strain-specific gene families of *M. tengchongensis* Ric-A were enriched (*p*-value < 0.05) in methyltransferase-related functions (Figure 5); these genes are probably involved in arsenic detoxification by catalyzing the formation of volatile trimethylarsine from arsenite (Qin et al., 2006; Ai et al., 2017).

**FIGURE 6:**
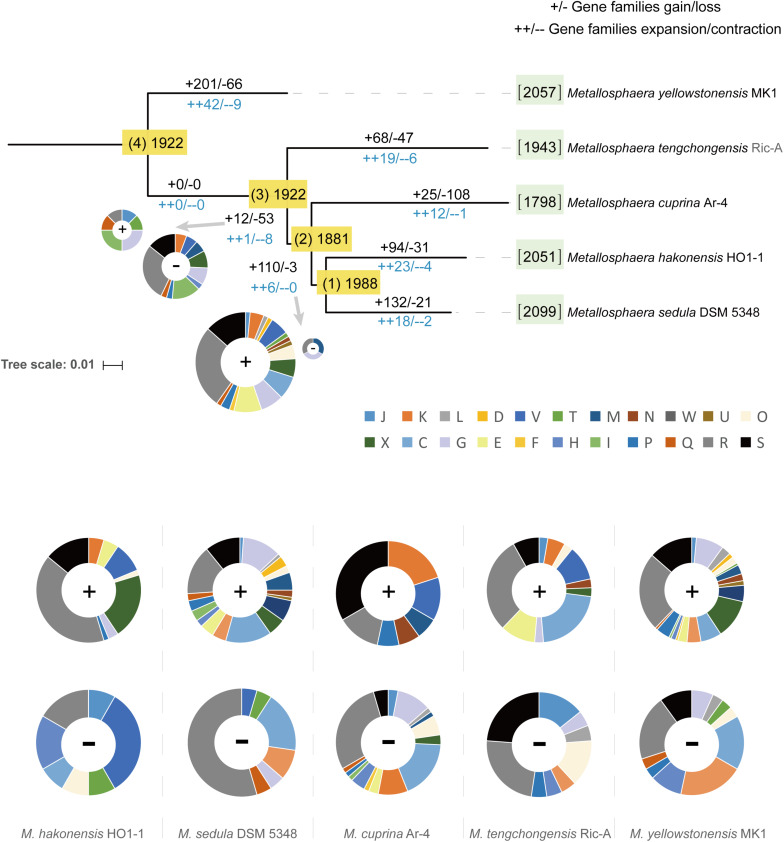
Ancestral genome content reconstruction of *Metallosphaera* species with COUNT software. The numbers of gene families of each strain were shown in brackets before the names of strains. The numbers of gain and loss events were marked at each lineage of the tree. “+”s represent gain events and “–”s represent loss events. The pie chart shows the numbers of gained genes by COG categories. A list of gained and lost genes for the *Metallosphaera*-type strains, and nodes 1 and 2 are shown in Supplementary Table S4. COG categories description (J, translation, ribosomal structure and biogenesis; K, transcription; L, replication, recombination and repair; D, cell cycle control, cell division, chromosome partitioning; V, defense mechanisms; T, signal transduction mechanisms; M, cell wall/membrane/envelope biogenesis; N, cell motility; U, intracellular trafficking, secretion, and vesicular transport; O, posttranslational modification, protein turnover, chaperones; X, mobilome: prophages, transposons; C, energy production and conversion; G, carbohydrate transport and metabolism; E, amino acid transport and metabolism; F, nucleotide transport and metabolism; H, coenzyme transport and metabolism; I, lipid transport and metabolism; P, inorganic ion transport and metabolism; Q, secondary metabolites biosynthesis, transport and catabolism; R, general function prediction only; S, function unknown).

To decipher the evolutionary histories of the *Metallosphaera* species, gene family gain, loss, expansion, and contraction events were predicted by mapping the identified gene families onto the core-gene tree (Figure 6). A large number of gene family gain events occurred at node 1, accounting for ∼6% of gene families, and at the branches of *M. yellowstonensis* and *M. sedula*, accounting for ∼10 and 6% of gene families, respectively. By means of gene acquisition, members of *Metallosphaera* have largely expanded their genetic diversity, resulting in functional divergence, which was similar to other Archaea (Brügger et al., 2002). Of gene families undergoing gain events, about half of them were poorly characterized, and a considerable proportion of them were related to COG (X) Mobilome: prophages, transposons (∼6%), COG (K) Transcription (∼3%), and COG (V) Defense mechanisms (∼3%, which mostly were associated with CRISPR-Cas system) (Figure 6). Several sulfate assimilation and archaeal flagella biosynthesis-related genes were gained at the branches of *M. yellowstonensis* and *M. sedula*, indicating that sulfate assimilation and flagella biosynthesis were derived features of these strains (Supplementary Table S4). Several DNA damage repair genes (e.g., *uve* and *spl*) that helped maintain DNA fidelity were gained at the branches of *M. hakonensis*, *M. tengchongensis*, *M. sedula*, *M. yellowstonensis*, and node 1, and genes associated with EPS synthesis (e.g., *rfa* and *gal*) that enhanced colonization were gained in the branches of *M. sedula* and *M. yellowstonensis* (Supplementary Table S4). Several oxidoreductase encoding genes that associated with carbohydrate metabolism (e.g., *porAB* and *acoAB*) were gained at the branches of *M. sedula* and *M. tengchongensis*. A few of oxidoreductase genes related to aromatic compound degradation (e.g., *hca* and *nfn*) were gained at the branches of *M. yellowstonensis*, *M. sedula*, and node 2. Genes encoding for putative type I CODH were expanded in *M. sedula* but contracted in *M. yellowstonensis* (Supplementary Table S4). Sulfocyanin encoding genes were expanded in *M. hakonensis*, *M. tengchongensis*, and Heme/copper-type cytochrome/quinol oxidase encoding genes associated with sulfur and iron oxidation (Supplementary Table S4) were expanded in the branches of node 1 and *M. yellowstonensis*. Indicating their importance in niche adaption, several genes involved in nitrogen transport and metabolism were expanded in several species: ammonia permease encoding gene *amtB* in *M. cuprina* and *M. tengchongensis*, nitrite reductase encoding gene *nirD* in *M. hakonensis* and *M. sedula*, and adenosylmethionine decarboxylase encoding gene *speD* involved in polyamine biosynthesis in *M. yellowstonensis.* However, the urease encoding gene operon *ureABCDEF* was lost at the branch of *M. yellowstonensis* (Supplementary Table S4). In contrast to conspicuous gene acquisitions in other *Metallosphaera* strains, gene family loss events occurred frequently in *M. cuprina* taking up ∼6% of gene families, which mostly compromised genes belonging to COGs Energy production and conversion (C) and Carbohydrate transport and metabolism (G) such as permease of fucose, sugar, arabinose, dehydrogenase of succinate, tartrate, malate/L-lactate, aldehyde and altronate, and oxidase of sulfite (Supplementary Table S4). Genomic streamlining in adaptation to the acidic, thermal, and oligotrophic environment may be the main reason for gene family losses in *M. cuprina*; previous studies showed that growth temperature was negatively correlated with genome size in bacteria (Sabath et al., 2013) and that deletion of dispensable sequences from bacterial genomes led to dose-dependent growth (Kurokawa et al., 2016; Zhang et al., 2017; Ren et al., 2019).

### Selective Pressure Analyses

**FIGURE 7:**
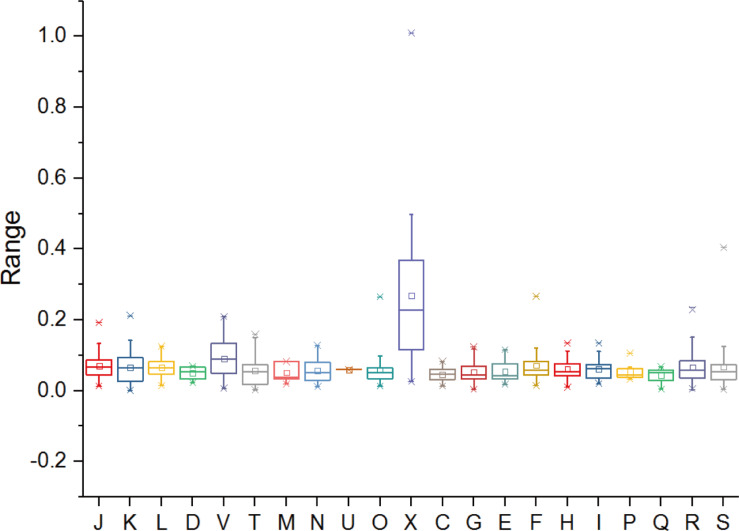
Distribution of selection pressures (indicated by values of dN/dS) on various gene families of *Metallosphaera* spp. based on COG categories. dN, non-synonymous (amino acid-changing) substitutions; dS, synonymous (amino acid-unchanging) substitutions. dN:dS < 1 indicates the action of purifying selection (negative selection), which purges deleterious mutations to conserve the protein structure and function (Carretero-Paulet et al., 2015).

Functions undergoing rapid evolution can be distinguished, taking into account the functional categories and selective pressure (Carretero-Paulet et al., 2015; Zhong et al., 2018). To gain insight into the conservation and evolution of various gene families in *Metallosphaera*, evolutionary pressure on each gene family that contained at least three non-identical sequences in five species of *Metallosphaera* was measured by calculating global substitution rates (dN/dS) of non-synonymous (dN) to synonymous (dS), as well as numbers of mutation sites under significant negative or positive selection in each gene family. Results indicated that there was pervasive strong purifying (negative) selection (dN/dS < 1) acting on the gene families of *Metallosphaera* with no gene families showing positive selection (dN/dS > 1), among them 86.6% gene families showing a dN/dS ratio lower than 0.1 (Figure 7 and Supplementary Table S5), which emphasized purifying selection contributing largely to the long-term stability of *Metallosphaera* genomes by removing deleterious mutations. Gene families of *Metallosphaera* exhibit different degrees of purifying selection pressure as shown by COG annotation. Genes undergoing the strongest purifying selection were those related to Transcription (K), indicating that these functions were highly conserved, while genes related to mobilome: prophages, transposons (X) and Defense mechanisms (V) were under weaker purifying pressure since they showed relatively higher dN/dS ratios and more sites under diversifying (positive) selection, which indicated that these genes could gain specific adaptive mutations associated with the acquisition of new or adaptive functions (Figure 7 and Supplementary Table S5).

### Genome Expansion Through HGT

**FIGURE 8:**
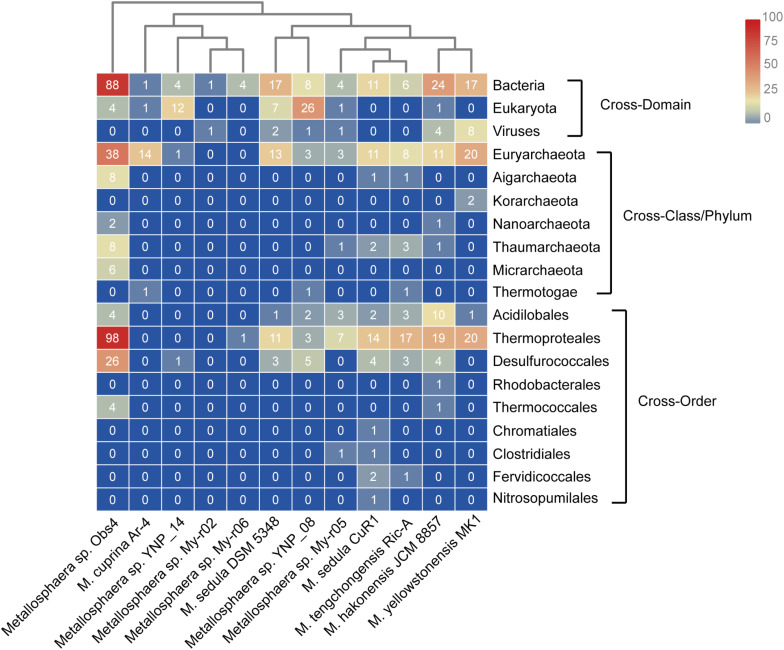
Heat map showing the number and distribution of predicted horizontally transferred genes in different *Metallosphaera* strains and their predicted donors (right).

Horizontal gene transfer is one of the important engines that drive genomic diversity and adaptive evolution of microbes, especially in the case of those that inhabit extreme environments (Olga et al., 2009; Zhang et al., 2017; Li et al., 2019), which in this case also contributed considerably to genome contents of *Metallosphaera*. Based on COG annotation, the transferred genes in *Metallosphaera* spp. comprised mostly defensive and metabolic functions, with approximately 6.7% defense mechanisms (V), 6.4% energy production and conversion (C), 6.4% carbohydrate transport and metabolism (G), 4.9% amino acid transport and metabolism (E), 3.9% coenzyme transport and metabolism (H), 3.2% inorganic ion transport and metabolism (P), 2.1% lipid transport and metabolism (I), and 1.1% secondary metabolites biosynthesis, transport, and catabolism (Q) (Supplementary Figure S13). Informational proteins such as ribosomal proteins and RNA processing proteins experienced fewer HGT events in comparison with other gene families (Supplementary Figure S13). Most HGT events appeared to be acquired from the same domain (Archaea), which is typical among the identified HGTs (Figure 8 and Supplementary Table S6). Bacteria also contributed largely to the emerging genetic diversity of *Metallosphaera* through cross-domain HGT, and acquired genes such as hydrogenase and benzoate/toluate dioxygenase also appeared to facilitate adaption to different niches, resulting in the functional divergence within the genus of *Metallosphaera* (Figure 8 and Supplementary Table S6). However, it should be noted that though metagenome-assembled genomes (MAGs) included in this study have supplemented available genomic data of *Metallosphaera* and expanded sample size, these MAGs may contain contamination generated during the binning process. Thus, the results of MAGs should be interpreted carefully.

## Conclusion

In this study, the genome of a *Metallosphaera* species (*M. tengchongensis*) was comparatively analyzed with 18 other genomes from *Metallosphaera* strains to enrich our understanding of the genetic traits, metabolism, and environmental adaption mechanism of the *Metallosphaera* and shed light on their evolutionary history. *Metallosphaera* microbes were widely found in sulfur-rich and metal-burdened environments, and all *Metallosphaera* strains analyzed in this study contained sulfur and iron oxidation genes. However, the APS pathway was only detected in *M. sedula* and *M. yellowstonensis*, and certain subunits of the *fox* cluster were lost in *M. cuprina*. Gene deficiency of SOR hinted that new unusual enzymes might undertake the S^0^ oxidation role. Complete TCA cycles and ED pathways coexisted with HP/HB and DC/HB cycles in all genomes of these strains and supported their heterotrophic, autotrophic, and mixotrophic growth modes. The genes for assimilatory nitrate reduction were present in all genomes of 19 strains; however, a complete dissimilatory nitrate reductase gene cluster was only found in *M. yellowstonensis*, which demonstrated that *Metallosphaera* species and strains have different abilities to use inorganic nitrogen. Genes encoding for flagellin and flagella accessory proteins were only detected in strains of *M. sedula*, *M. yellowstonensis*, *Metallosphaera* sp. Obs4, *Metallosphaera* sp. UBA165, and *Metallosphaera* sp. My_r06. We also found that functions related to assimilatory sulfur metabolism and cysteine biosynthesis associated with ROS reduction were significantly enriched in accessory gene families. Evolutionary analyses showed that massive gene family gain events occurred at the branches of *M. yellowstonensis* and *M. sedula*, whereas considerable gene family loss events occurred in *M. cuprina* and pervasive strong purifying selection was found acting on the gene families of *Metallosphaera*. We also found that HGT played an important role in shaping the genetic and functional diversity of *Metallosphaera*. These findings provide a data basis for subsequent studies of metabolism and environmental adaption mechanisms in thermophilic Archaea, and for strategies to design cellular biocatalysts for the biomining process.

## Data Availability Statement

The datasets generated for this study can be found in the genome sequence of Metallosphaera tengchongensis strain Ric-A has been deposited at JGI IMG-ER database under the IMG Taxon OID 2821472399, and Genbank database under accession number CP049074.

## Author Contributions

C-YJ, S-JL, and HY designed and coordinated the study. PW, LZL, and LJL performed the bioinformatics analysis. YQ, ZL, and XL carried out the experiments and interpreted data for the work. PW and LZL wrote the manuscript. C-YJ and S-JL edited the manuscript. All authors contributed to the article and approved the submitted version.

## Conflict of Interest

The authors declare that the research was conducted in the absence of any commercial or financial relationships that could be construed as a potential conflict of interest.

## Footnotes

1 https://jgi.doe.gov/user-programs/pmo-overview/protocols-sample-preparation-information/

2 https://www.pacb.com/support/software-downloads/

3 https://pypi.org/project/pyani/

4 http://www-is.biotoul.fr

5 https://www.pathogenomics.sfu.ca/islandviewer/

6 http://phaster.ca/

7 https://crisprcas.i2bc.paris-saclay.fr/CrisprCasFinder/Index

8 https://orthovenn2.bioinfotoolkits.net/home

9 http://www.datamonkey.org/fel/

## Supplementary Material

FIGURE S1 Phylogenetic tree of *Metallosphaera* spp. with **(A)** Maximum Likelihood (ML) method; **(B)** Minimum Evolution (ME) method; **(C)** UPGMA method; **(D)** Maximum Parsimony (MP) method based on 16S rRNA sequences. Bootstrap values are indicated at each node based on a total of 1000 bootstrap replicates.

FIGURE S2 Whole genome alignment with Mauve showing syntenic blocks between type strains *M. tengchongensis* Ric-A, *M. cuprina* Ar-4, *M. sedula* DSM 5348, and *M. hakonensis* HO1-1 (from top to bottom).

FIGURE S3 BlastN-based whole genome comparison of strains *M. cuprina* Ar-4, *M. hakonensis* HO1-1, *Metallosphaera* sp. My-r06,*M. sedula* DSM 5348, *M. tengchongensis* Ric-A, and *M. yellowstonensis* MK1 (from inside to outside) with BRIG, and these strains were used as reference, respectively. GC content and GC skew (inner rings 1 and 2) of each genome were also indicated.

FIGURE S4 Phylogenetic tree of *Metallosphaera* spp. rooted by *Encephalitozoon cuniculi* GB M1 and *Prochlorococcus marinus* MIT 9301 based on whole genome sequences using CVTree 3.

FIGURE S5 Phylogenetic tree of *Metallosphaera* spp. and other members of *Sulfolobales* based on whole genome sequences using CVTree 3.

FIGURE S6 Heat map showing the distribution and number of genes encoding putative hydrogenase related proteins in different *Metallosphaera* strains.

FIGURE S7 Heat map showing the distribution and numbers of genes encoding putative proteins involved in iron oxidation in different *Metallosphaera* strains.

FIGURE S8 Heat map showing the distribution and numbers of genes encoding carbohydrate active enzymes (CAZymes) in different *Metallosphaera* strains. Abbreviations in the chart: *AA*, auxiliary activities proteins; *CBM*, carbohydrate-binding molecules; *CE*, carbohydrate esterases; *GH*, glycoside hydrolases; *GT*, glycosyltransferases; *PL*, polysaccharide lyases.

FIGURE S9 Heat map showing the distribution and numbers of genes encoding putative enzymes involved in carbon metabolism in different *Metallosphaera* strains. Abbreviation and KO term descriptions in the chart: *CoxL/M/S*, Carbon monoxide dehydrogenase large/medium/small chain; *GalE*, UDP-glucose 4-epimerase; *RfbA*, d-TDP-glucose pyrophosphorylase; *RfbB*, d-TDP-glucose 4;6-dehydratase; *RfbC*, d-TDP-4-dehydrorhamnose 3;5-epimerase; *RfbD*, d-TDP-4-dehydrorhamnose reductase; *K01895*, acetyl-CoA synthetase; *K00172*, pyruvate ferredoxin oxidoreductase gamma subunit; *K00626*, acetyl-CoA C-acetyltransferase; *K01681* aconitate hydratase; *K01007*, pyruvate; water dikinase (EC:2.7.9.2); *K01964* acetyl-CoA/propionyl-CoA carboxylase (EC:6.4.1.2); *K15016*, enoyl-CoA hydratase; *K15017*, malonyl-CoA/succinyl-CoA reductase; *K01595*, phosphoenolpyruvate carboxylase; *K15039* 3-hydroxypropionate dehydrogenase (NADP+) (EC:1.1.1.298); *K15018*, 3-hydroxypropionyl-coenzyme A synthetase; *K15019* 3-hydroxypropionyl-coenzyme A dehydratase; *K14534*, -hydroxybutyryl-CoA dehydratase; *K00031*, isocitrate dehydrogenase; *K00024*, malate dehydrogenase; *K15020*, acryloyl-coenzyme A reductase; *K14466*, 4-hydroxybutyrate-CoA ligase; *K05606*, methylmalonyl-CoA; *K00175*, 2-oxoglutarate/2-oxoacid ferredoxin oxidoreductase; *K01678*, fumarate hydratase; *K01848* methylmalonyl-CoA mutase; *K18859*, succinate dehydrogenase/fumarate reductase; *K01902*, succinyl-CoA synthetase.

FIGURE S10 Heat map showing the distribution and numbers of genes encoding putative proteins involved in nitrogen metabolism in different *Metallosphaera* strains. Abbreviation descriptions in the chart: *GdhA*, glutamate dehydrogenase [NAD(P)+] (EC 1.4.1.3); *GlnA*, glutamine synthetase (EC 6.3.1.2); *CarA*, carbamoyl-phosphate synthase small subunit (EC 6.3.5.5); *CarB*, carbamoyl-phosphate synthase large subunit (EC 6.3.5.5); *NarG*, nitrate reductase/nitrite oxidoreductase, alpha subunit (EC 1.7.5.1; 1.7.99.4); *NarH*, nitrate reductase/nitrite oxidoreductase, beta subunit (EC 1.7.5.1; 1.7.99.4); *NarJ*, nitrate reductase delta subunit; *Nar*I, nitrate reductase gamma subunit (EC 1.7.5.1; 1.7.99.4); *Nrt*, nitrate/nitrite transporter; *Amt*, ammonium transporter; *UreAB*, *UreCD*, urease functional subunits; *UreE*, *UreF*, *UreG*, urease accessory proteins; *ArgH*, argininosuccinate lyase; *ArgI*, ornithine carbamoyltransferase; *ArcA*, arginine decarboxylase; *ArgG*, argininosuccinate synthase; *SpeB*, agmatinase; *SpeD*, S-adenosylmethionine decarboxylase; *SpeE*, spermidine synthase.

FIGURE S11 Heat map showing patterns of the distribution and numbers of genes encoding putative proteins involved in heavy metal resistance in different *Metallosphaera* strains.

FIGURE S12 Heat map showing patterns of the distribution and numbers of genes encoding putative proteins involved in flagella biosynthesis in different *Metallosphaera* strains.

FIGURE S13 The COG proportions of predicted horizontal transferred genes in *Metallosphaera* spp.

TABLE S1 Average nucleotide identity (ANI) based on whole genome alignments of *Metallosphaera* spp.

TABLE S2 Statistics of genomic islands in the genomes of *Metallosphaera* spp.

TABLE S3 Statistics of Mobile genetic elements (MGEs) (A), prophages and prophage remnants (B), Transposons (C), CRISPR-Cas related sequences (D) present in the genomes of *Metallosphaera* spp.

TABLE S4 Gene families undergoing gain (A), loss (B), expansion (C), and contraction (D) events in *Metallosphaera* spp. detected with COUNT.

TABLE S5 Selection pressure on gene families of Metallosphaera spp.

TABLE S6 Putative horizontally transferred genes of *Metallosphaera* spp.
