# Supplementary material for: Comparative Genomic Analysis Reveals the Metabolism and Evolution of the Thermophilic Archaeal Genus Metallosphaera
Source: Front Microbiol. 2020 Jun 19;11:1192. doi: 10.3389/fmicb.2020.01192 (PMC7325606; doi:10.3389/fmicb.2020.01192)
Supplement: FIGURE S1 — Phylogenetic tree of Metallosphaera spp. with (A) Maximum Likelihood (ML) method; (B) Minimum Evolution (ME) method; (C) UPGMA method; (D) Maximum Parsimony (MP) method based on 16S rRNA sequences. Bootstrap values are indicated at each node based on a total of 1000 bootstrap replicates. [file Data_Sheet_1.PDF]

Comparative Genomic Analysis Reveals the Metabolism and Evolution of the Thermophilic Archaeal Genus *Metallosphaera*

Pei Wang[1,2,§], LiangZhi Li[3,4,§], YaLing Qing[1,2], ZongLin Liang[1,2], XiuTong Li[1,2], HuaQun Yin[3,4], LiJun Liu[5], Shuang-Jiang Liu[1,2]* and Cheng-Ying Jiang[1,2]*

[1]State Key Laboratory of Microbial Resources, Institute of Microbiology, Chinese Academy of Sciences, Beijing 100101, China

[2]University of Chinese Academy of Sciences, Beijing 100049, China

[3]School of Resources Processing and Bioengineering, Central South University, Changsha, China

[4]Key Laboratory of Biometallurgy of Ministry of Education, Central South University, Changsha

[5]Department of Pathogen Biology, School of Basic Medical Science, Xi'an Medical University, Xi'an 710021, Shaanxi Province, China

[§] P. Wang. and LZ. Li. contributed equally

*** Correspondence:**

Cheng-Ying Jiang

jiangcy@im.ac.cn

Shuang-Jiang Liu

liusj@im.ac.cn

Supplementary Figure

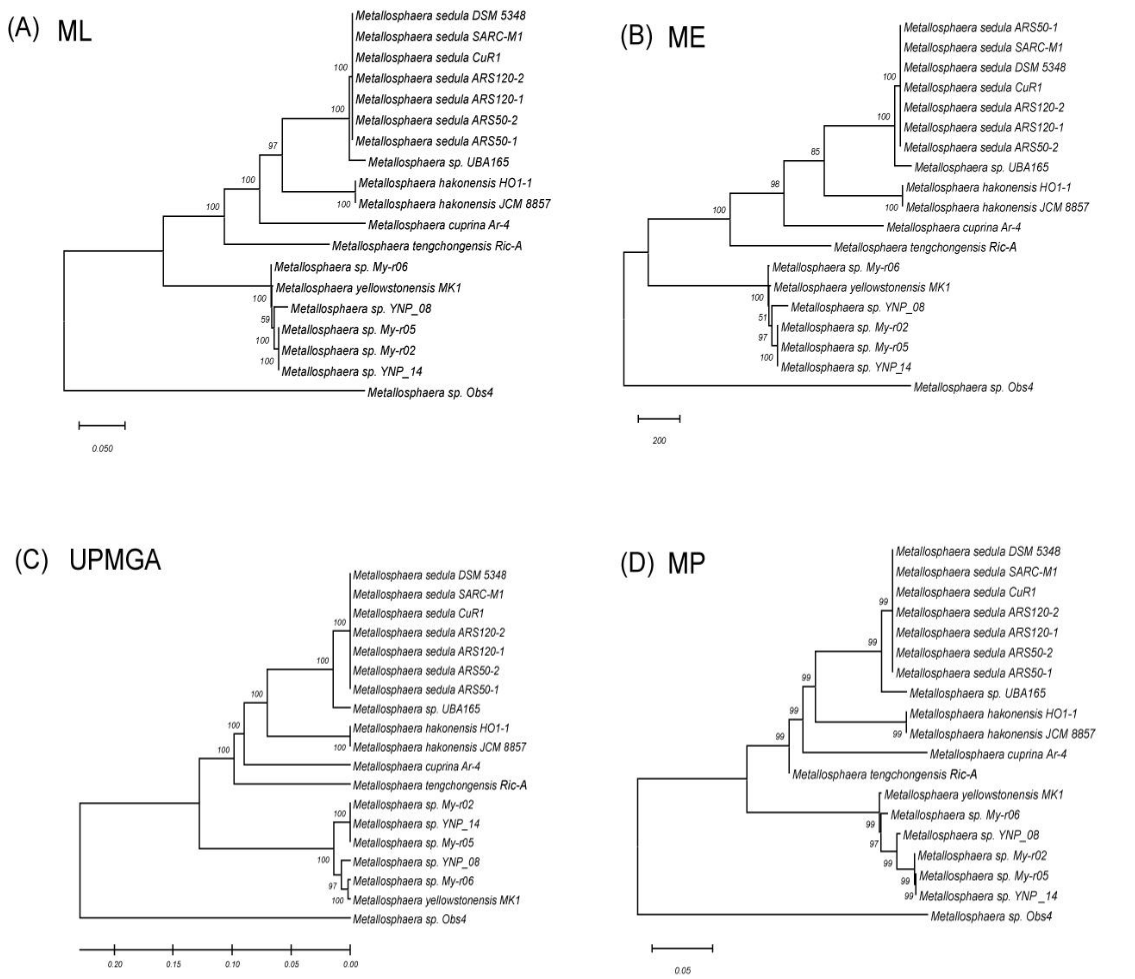

Fig. S1

Phylogenetic tree of *Metallosphaera* spp. with (A) Maximum Likelihood (ML) method; (B) Minimum Evolution (ME) method; (C) UPGMA method; (D) Maximum Parsimony (MP) method based on 16S rRNA sequences. Bootstrap values are indicated at each node based on a total of 1,000 bootstrap replicates.

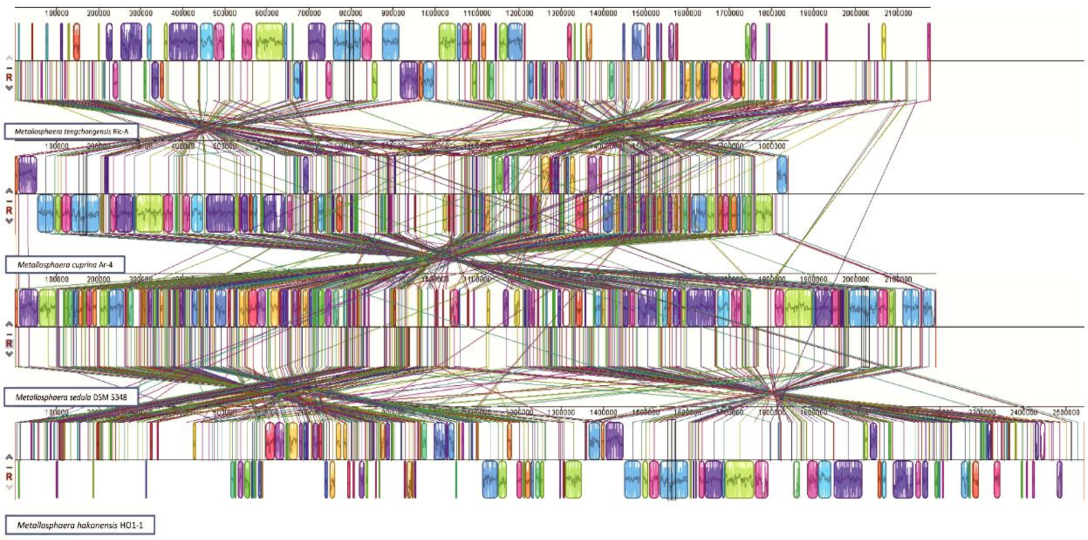

Fig. S2

Whole genome alignment with Mauve showing syntenic blocks between type strains *M. tengchongensis* Ric-A, *M. cuprina* Ar-4, *M. sedula* DSM 5348, and *M. hakonensis* HO1-1 (from top to bottom).

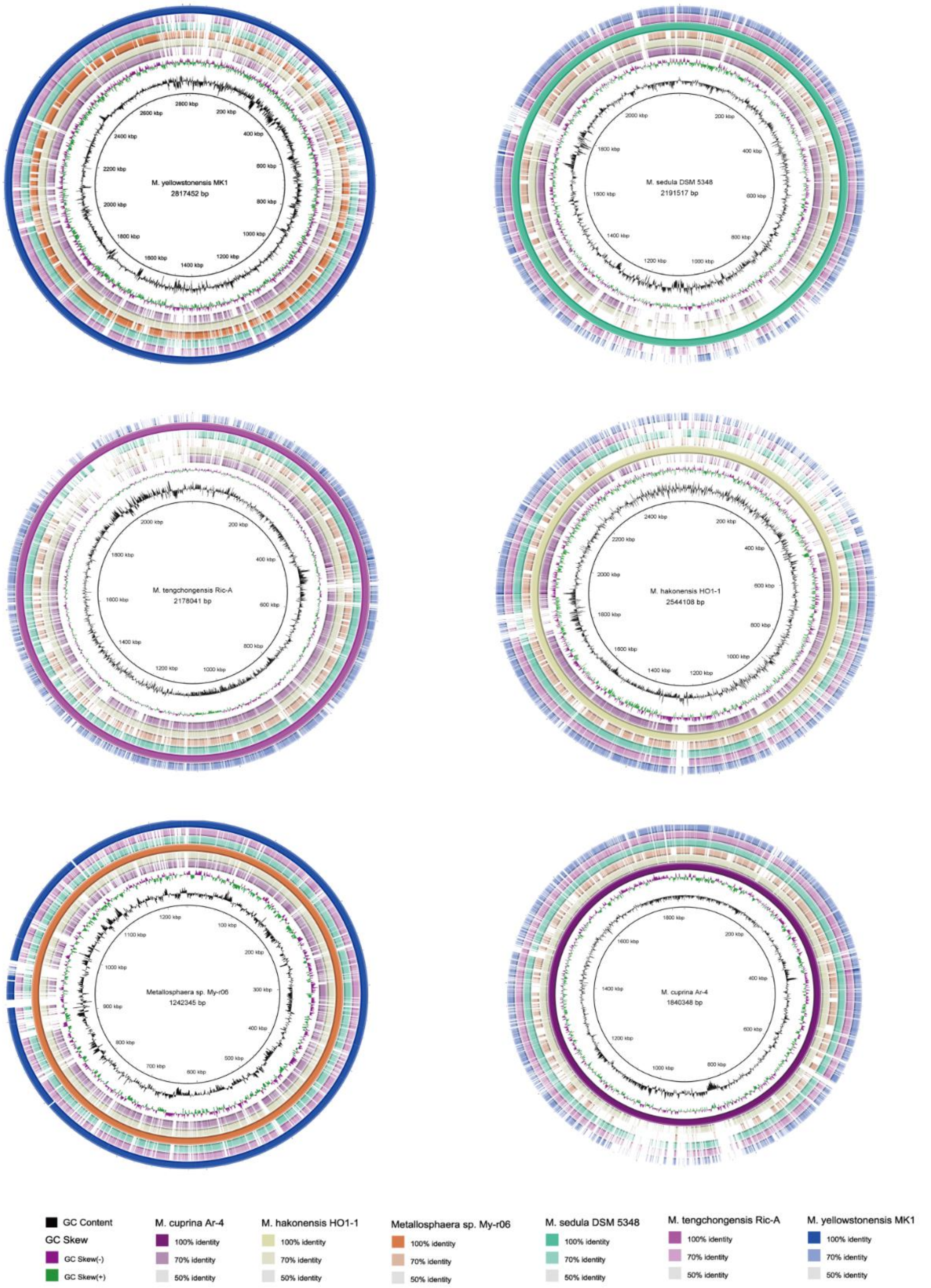

Fig. S3

BlastN-based whole genome comparison of strains *M. cuprina* Ar-4, *M. hakonensis* HO1-1, *Metallosphaera* sp. My-r06，*M. sedula* DSM 5348, *M. tengchongensis* Ric-A, and *M. yellowstonensis* MK1 (from inside to outside) with BRIG, and these strains were used as reference, respectively. GC content and GC skew (inner ring 1 and 2) of each genome were also indicated.

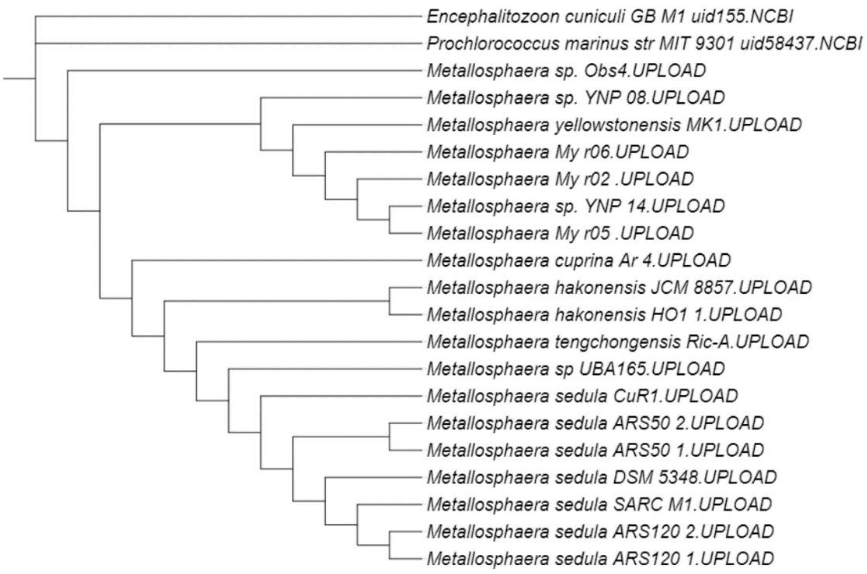

Fig. S4

Phylogenetic tree of *Metallosphaera* spp. rooted by *Encephalitozoon cuniculi* GB M1 and *Prochlorococcus marinus* MIT 9301 based on whole genome sequences using CVTree 3.

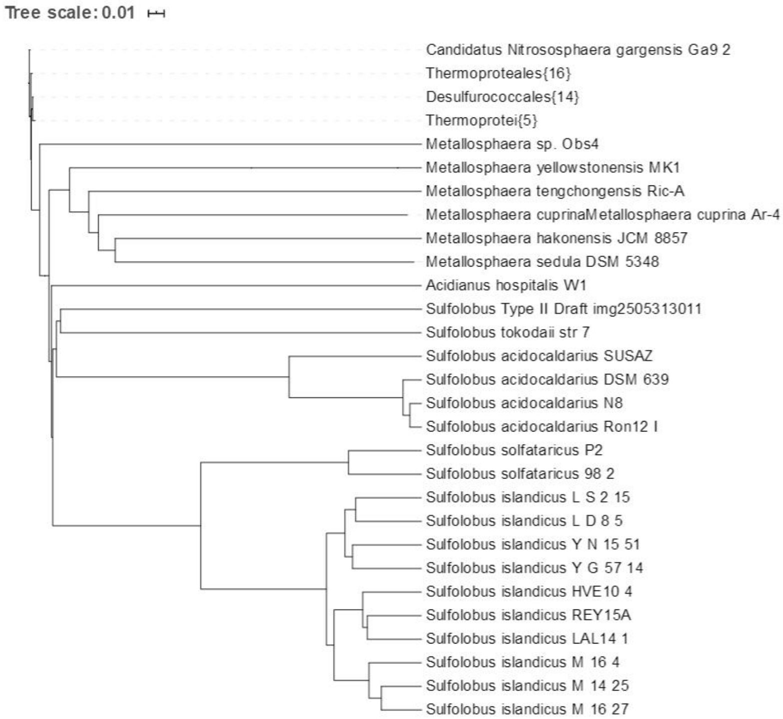

Fig. S5

Phylogenetic tree of *Metallosphaera* spp. and other members of Sulfolobales based on whole genome sequences using CVTree 3.

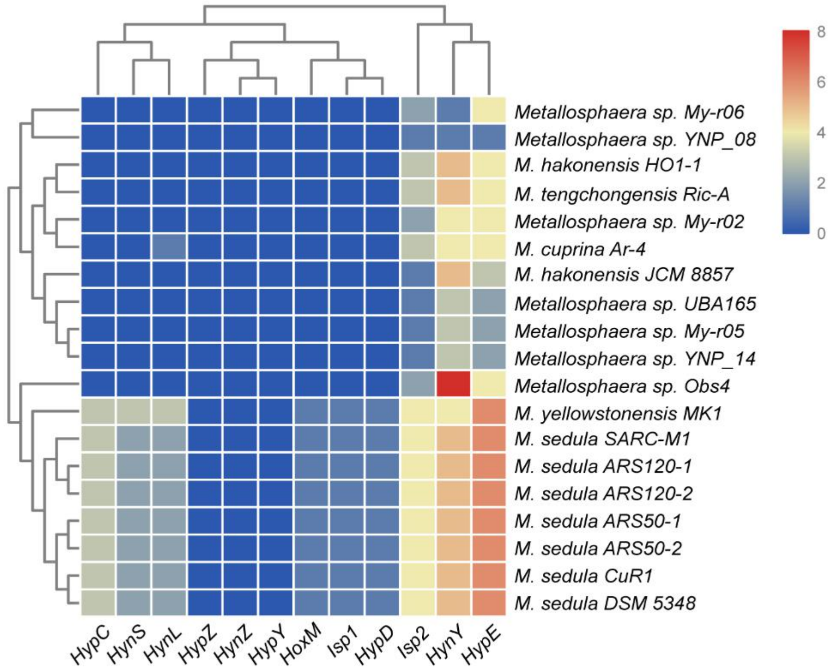

Fig. S6

Heat map showing the distribution and number of genes encoding putative hydrogenase related proteins in different *Metallosphaera* strains.

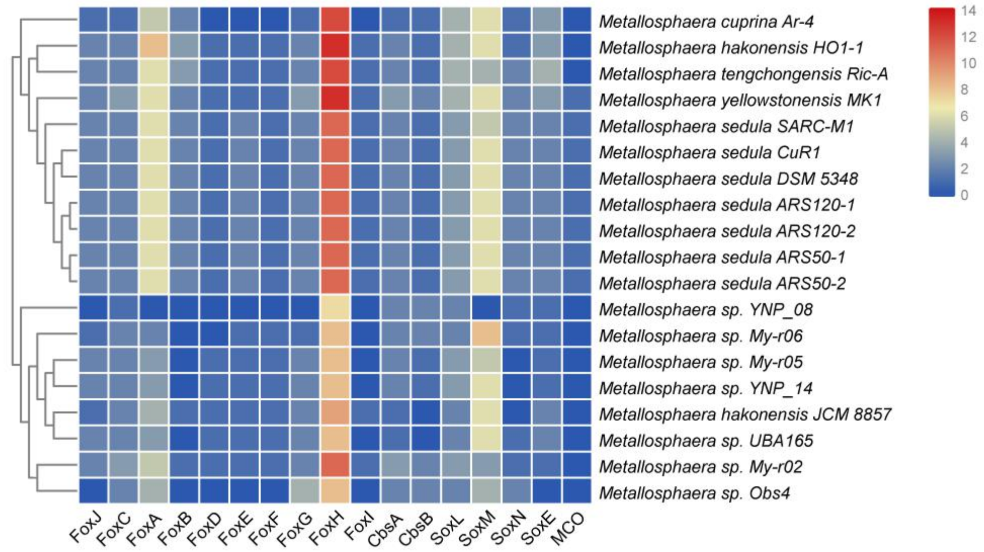

Fig. S7

Heat map showing the distribution and numbers of genes encoding putative proteins involved in iron oxidation in different *Metallosphaera* strains.

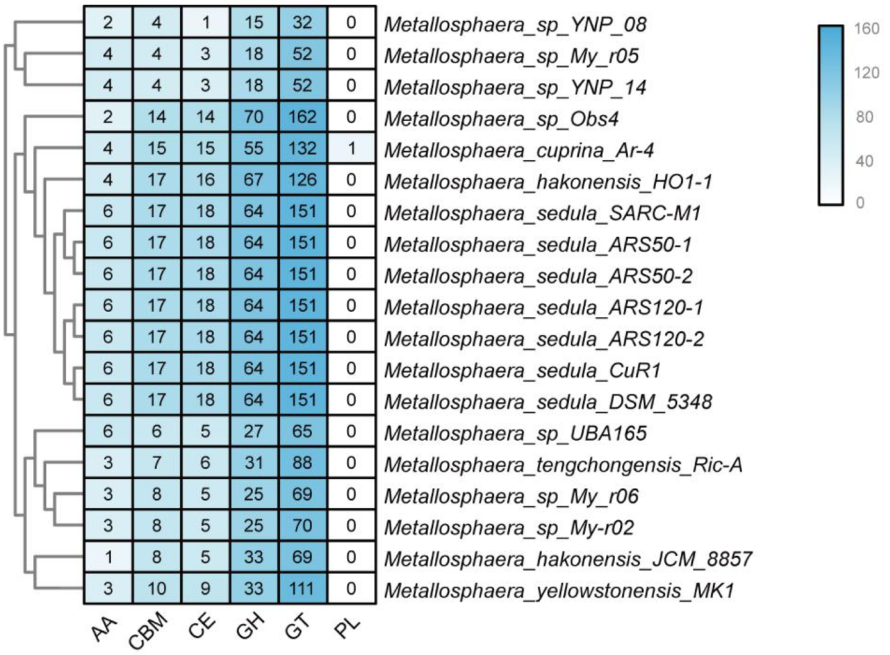

Fig. S8

Heat map showing the distribution and numbers of genes encoding carbohydrate active enzymes (CAZymes) in different *Metallosphaera* strains. Abbreviations in the chart: *AA,* auxiliary activities proteins; *CBM,* carbohydrate-binding molecules; *CE*, carbohydrate esterases; *GH,* glycoside hydrolases; *GT,* glycosyltransferases; *PL,* polysaccharide lyases.

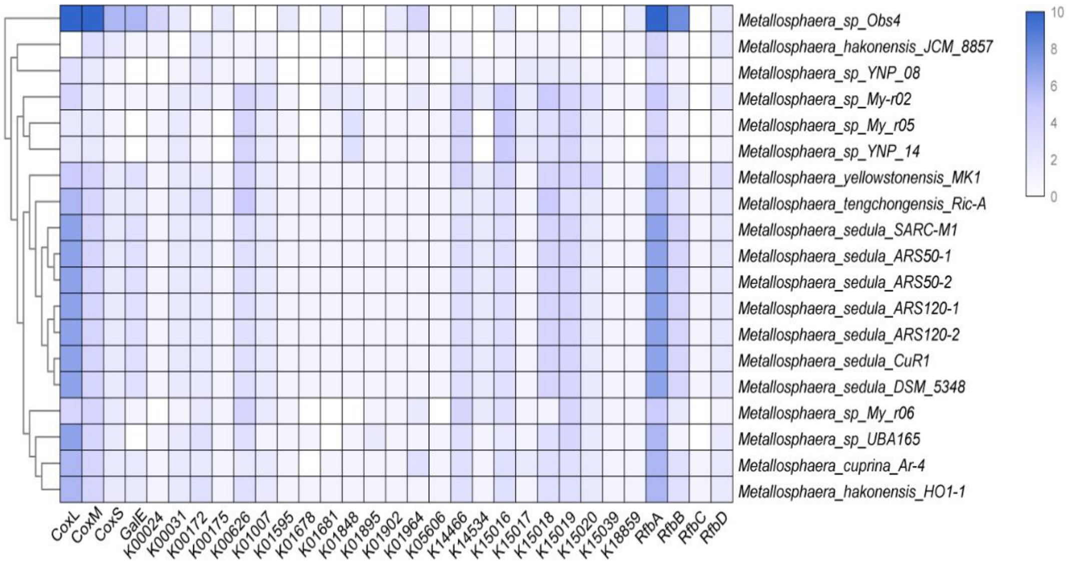

Fig. S9

Heat map showing the distribution and numbers of genes encoding putative enzymes involved in carbon metabolism in different *Metallosphaera* strains. Abbreviation and KO term descriptions in the chart: *CoxL/M/S,* Carbon monoxide dehydrogenase large/

medium/small chain; *GalE,* UDP-glucose 4-epimerase; *RfbA,* d-TDP-glucose pyrophosphorylase; *RfbB,* d-TDP-glucose 4;6-dehydratase; *RfbC,* d-TDP-4-dehydrorhamnose 3;5-epimerase; *RfbD,* d-TDP-4-dehydrorhamnose reductase; *K01895,* acetyl-CoA synthetase; *K00172,* pyruvate ferredoxin oxidoreductase gamma subunit; *K00626,* acetyl-CoA C-acetyltransferase; *K01681* aconitate hydratase; *K01007,* pyruvate; water dikinase [EC:2.7.9.2]; *K01964* acetyl-CoA/propionyl-CoA carboxylase [EC:6.4.1.2]; *K15016,* enoyl-CoA hydratase; *K15017,* malonyl-CoA/succinyl-CoA reductase; *K01595,* phosphoenolpyruvate carboxylase; *K15039* 3-hydroxypropionate dehydrogenase (NADP+) [EC:1.1.1.298]; *K15018,* 3-hydroxypropionyl-coenzyme A synthetase; *K15019* 3-hydroxypropionyl-coenzyme A dehydratase; *K14534*, -hydroxybutyryl-CoA dehydratase; *K00031,* isocitrate dehydrogenase ; *K00024,* malate dehydrogenase; *K15020* ,acryloyl-coenzyme A reductase; *K14466,* 4-hydroxybutyrate-CoA ligase; *K05606,* methylmalonyl-CoA; *K00175,* 2-oxoglutarate/2-oxoacid ferredoxin oxidoreductase; *K01678,* fumarate hydratase; *K01848* methylmalonyl-CoA mutase; *K18859,* succinate dehydrogenase / fumarate reductase; *K01902,* succinyl-CoA synthetase.

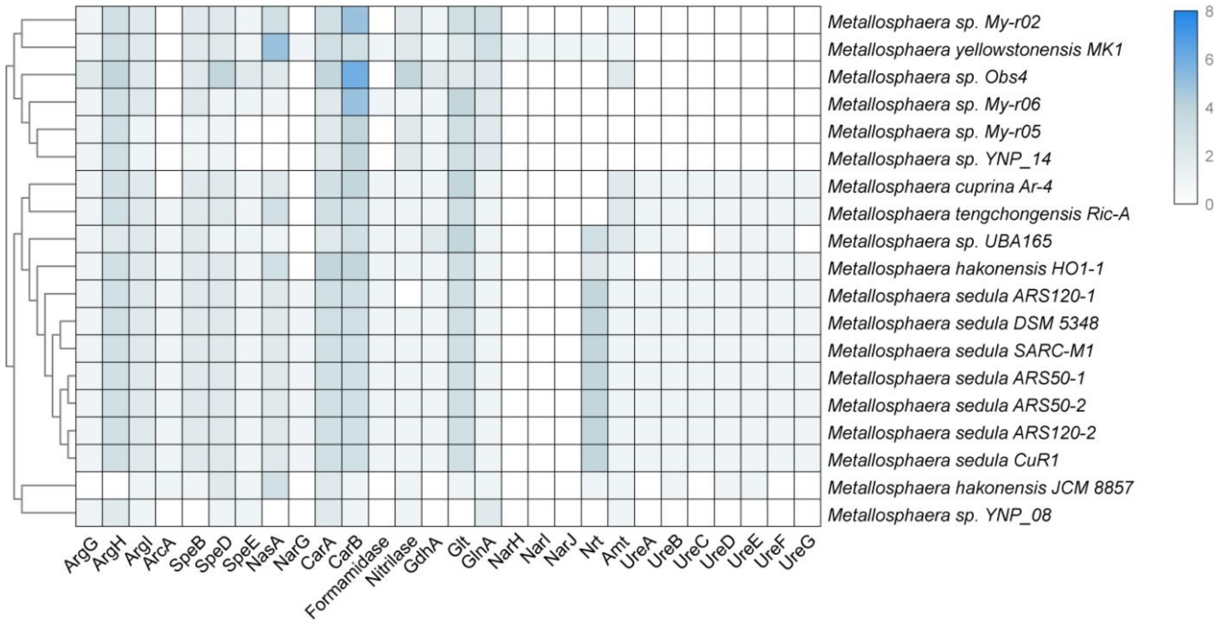

Fig. S10

Heat map showing the distribution and numbers of genes encoding putative proteins involved in nitrogen metabolism in different *Metallosphaera* strains. Abbreviation descriptions in the chart: *GdhA*, glutamate dehydrogenase (NAD(P)+) [EC 1.4.1.3]; *GlnA*, glutamine synthetase [EC 6.3.1.2]; *CarA*, carbamoyl-phosphate synthase small subunit [EC 6.3.5.5]; *CarB*, carbamoyl-phosphate synthase large subunit [EC 6.3.5.5]; *NarG*, nitrate reductase/nitrite oxidoreductase, alpha subunit [EC 1.7.5.1; 1.7.99.4];

NarH, nitrate reductase/nitrite oxidoreductase, beta subunit [EC 1.7.5.1; 1.7.99.4]; *NarJ*, nitrate reductase delta subunit; *NarI*, nitrate reductase gamma subunit [EC 1.7.5.1; 1.7.99.4]; *Nrt*, nitrate/nitrite transporter; *Amt*, ammonium transporter; *UreAB*, *UreCD,* urease functional subunits; *UreE, UreF, UreG*, urease accessory proteins; *ArgH*, argininosuccinate lyase; *ArgI*, ornithine carbamoyltransferase; *ArcA*, arginine decarboxylase; *ArgG,* argininosuccinate synthase; *SpeB,* agmatinase; *SpeD*, S-adenosylmethionine decarboxylase; *SpeE*, spermidine synthase.

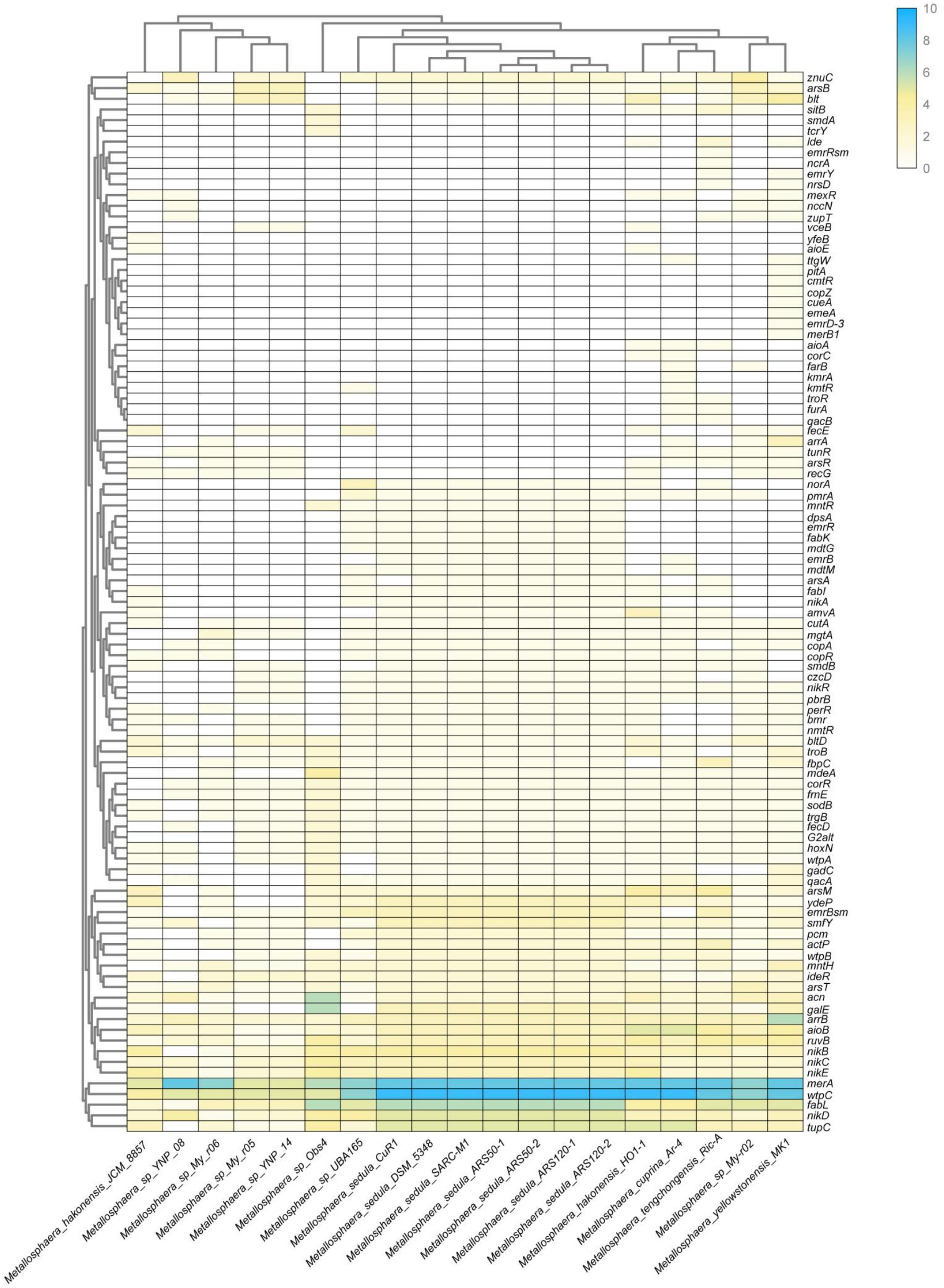

Fig. S11

Heat map showing patterns of the distribution and numbers of genes encoding putative proteins involved in heavy metal resistance in different *Metallosphaera* strains.

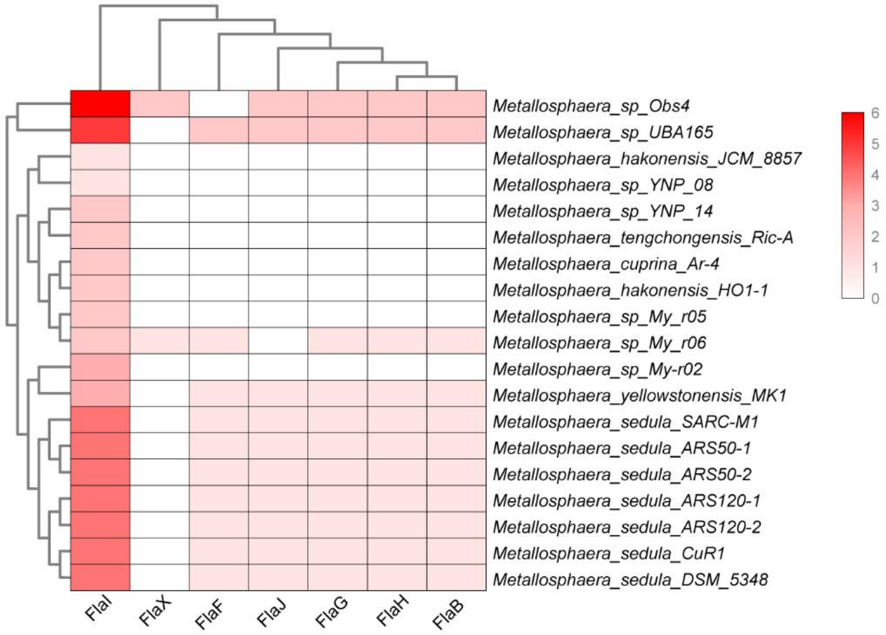

Fig. S12

Heat map showing patterns of the distribution and numbers of genes encoding putative proteins involved in flagella biosynthesis in different *Metallosphaera* strains.

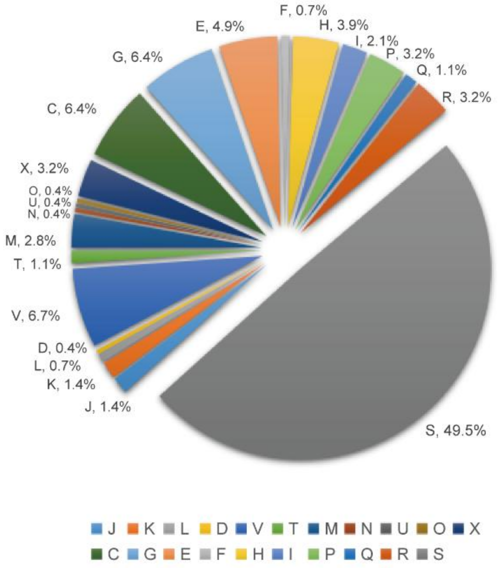

Fig. S13 The COG proportions of predicted horizontal transferred genes in *Metallosphaera* spp.
